# Activatable prodrug for controlled release of an antimicrobial peptide via the proteases overexpressed in *Candida albicans* and *Porphyromonas gingivalis*

**DOI:** 10.7150/thno.91165

**Published:** 2024-02-17

**Authors:** Lubna Amer, Maurice Retout, Jesse V. Jokerst

**Affiliations:** 1 Program in Materials Science and Engineering, University of California, San Diego, La Jolla, CA 92093, United States.; 2 Department of NanoEngineering, University of California, San Diego, La Jolla, CA 92093, United States.; 3 Department of Radiology, University of California, San Diego, La Jolla, CA 92093, United States.

**Keywords:** antimicrobial peptides, plasmonic nanoparticles, oral microorganisms, controlled assembly, protease sensing

## Abstract

*Candida albicans* and *Porphyromonas gingivalis* are prevalent in the subgingival area where the frequency of fungal colonization increases with periodontal disease. Candida's transition to a pathogenic state and its interaction with *P. gingivalis* exacerbate periodontal disease severity. However, current treatments for these infections differ, and combined therapy remains unexplored. This work is based on an antimicrobial peptide that is therapeutic and induces a color change in a nanoparticle reporter.

**Methods:** We built and characterized two enzyme-activatable prodrugs to treat and detect *C. albicans* and *P. gingivalis* via the controlled release of the antimicrobial peptide. The zwitterionic prodrug quenches the antimicrobial peptide's activity until activation by a protease inherent to the pathogens (SAP9 for *C. albicans* and RgpB for *P. gingivalis*). The toxicity of the intact prodrugs was evaluated against fungal, bacterial, and mammalian cells. Therapeutic efficacy was assessed through microscopy, disk diffusion, and viability assays, comparing the prodrug to the antimicrobial peptide alone. Finally, we developed a colorimetric detection system based on the aggregation of plasmonic nanoparticles.

**Results:** The intact prodrugs showed negligible toxicity to cells absent a protease trigger. The therapeutic impact of the prodrugs was comparable to that of the antimicrobial peptide alone, with a minimum inhibitory concentration of 3.1 - 16 µg/mL. The enzymatic detection system returned a detection limit of 10 nM with gold nanoparticles and 3 nM with silver nanoparticles.

**Conclusion:** This approach offers a convenient and selective protease sensing and protease-induced treatment mechanism based on bioinspired antimicrobial peptides.

## Introduction

The oral cavity contains the highest diversity of microorganisms among all human microbial environments and serves as a primary site of colonization and cross-kingdom interactions between fungi and bacteria 1, 2. Amongst the *Candida* genus, *Candida albicans* is the most commonly isolated species from the oral cavity and is responsible for most superficial and systemic fungal infections 3, 4. *C. albicans* is an opportunistic yeast-like fungus that colonizes human mucosal surfaces often becoming pathogenic in immunocompromised patients. This can lead to recurrent or even disseminated infections with high mortality rates 5. *Candida* interacts with other oral microbes to promote hyphal formation and enhance pathogenesis of both microorganisms 5-10. This pathogenic process is rooted in the colonization activity wherein *C. albicans* exploits an ability to switch morphology from yeast to hyphal form. Importantly, the hyphal form leads to the structured microbial biofilm with increased resistance to antimicrobial therapy 6, 11-14. Biofilms that extend below the gingival margin become subgingival plaque 15, 16. Here, pathogenic, gram-negative anaerobic bacteria such as *Porphyromonas gingivalis* can co-colonize the sulcus 15.

*P. gingivalis* is a keystone bacteria in periodontal disease and involves an inflammatory process with various degrees of severity ranging from readily curable gingivitis to irreversible periodontitis 15, 17-19. Data suggest that interactions between *C. albicans* and bacteria may modulate the clinical course of infection and negatively impact treatment 20. Likewise, the rate of colonization of *C. albicans* at subgingival sites is significantly higher in subjects with chronic periodontitis; the rate of candidiasis increases with higher isolation frequencies of *P. gingivalis* 21-23. However, treatment of *C. albicans* and *P. gingivalis* is quite different, and co-treatment has not yet been investigated in molecular detail 21. Treatment for *P. gingivalis*-based gingivitis is largely limited to lifestyle changes; thus, gingivitis relapses are common in susceptible patients leading to tooth loss and worse quality of life 24. Moreover, drug-resistant fungi are a growing public health threat with a mortality rate as high as 30% in the *Candida* family (*C. auris*); thus, improved therapies are clearly needed 3, 25.

Here, we report an activatable protease-responsive antimicrobial peptide as a novel therapeutic approach to treat and detect *C. albicans* and *P. gingivalis*. Antimicrobial peptides (**AMP**) are cationic, hydrophobic, and amphiphilic molecules and promising therapies against multi-drug resistant microorganisms. AMPs disrupt the cell membrane through electrostatic interactions with negatively charged components such as anionic lipopolysaccharides, phospholipids, and ergosterol 26, 27. However, AMPs can be non-specific and damage host cells 28, 29. Thus, we hypothesized that coating-activatable systems that are readily dependent on the pathogenesis of the microorganism would overcome this limitation 30. This is achieved by retaining the pharmacokinetic properties of the AMP and using peptide substrates that release it under direct exposure to a pathogenic protease.

More specifically, we tuned a prodrug for *C. albicans* to be responsive to secreted aspartyl proteases (**SAP**), which govern the transition from harmless commensal to disease-causing pathogen 22, 31-34. Similarly, gingipains (**gp**) are implicated in the pathogenesis of gingivitis and represent more than 85% of the proteolytic activity of *P. gingivalis* 35. Therefore, along with serving as a bacteria-presence signature, gingipains are also biomarkers of disease progression and development. We thus tuned a *P. gingivalis*- specific prodrug to respond to gingipains. Finally, we used the zwitterionic peptide-prodrugs to produce a colorimetric signal that reports the presence of *C. albicans* or *P. gingivalis* proteases 36, 37. This color change is based on precious metal nanoparticles and plasmonic coupling leading to a change in optical behavior in the visible region 35, 38-40. In the work below, we characterize the prodrugs, their toxicity profiles, their selectivity to fungi and bacteria *in vitro*, and finally, show the diagnostic performance of the sensor.

## Results and Discussion

**Scheme 1:**
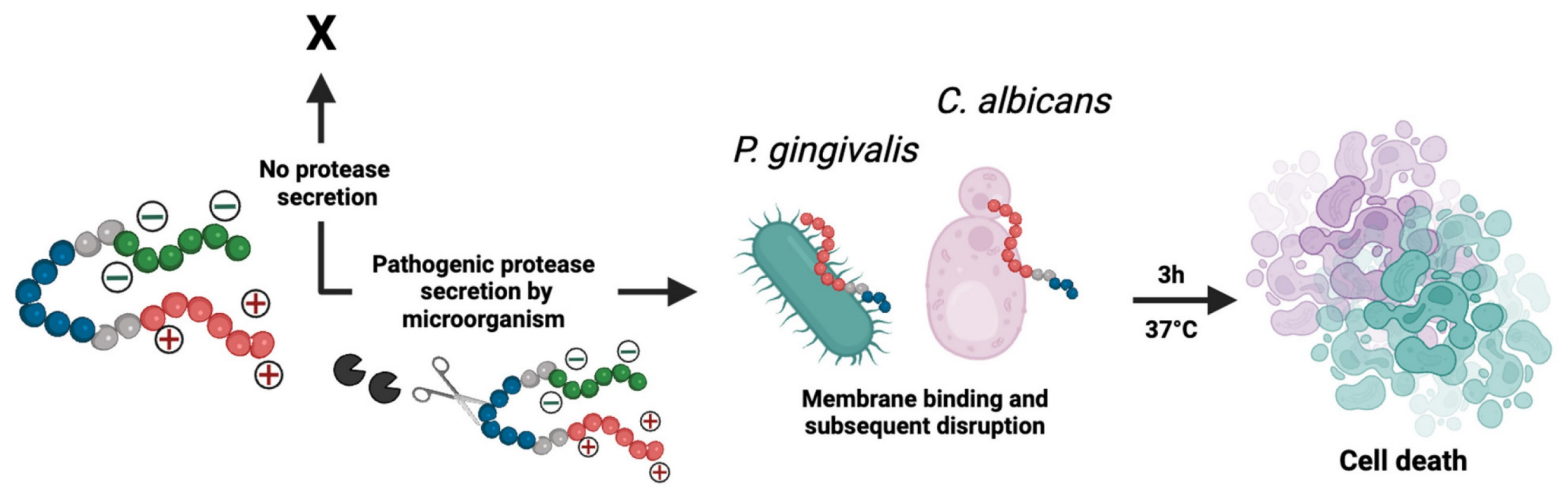
** Descriptive schematic of activatable therapy.** The three key components of the prodrugs: a therapy (P-113; **AKRHHGYKRKFH**) (red), a charge-shielding toxicity-quencher (DDDDDD) (green), and a recognition sequence for SAP9 (VKKK/DVVD) or RgpB (AGPR/ID) (blue). The net charge of the intact prodrug and its therapy is 0 and +5, respectively. An expression of pathogenic proteases induces cleavage of the prodrug, releasing the antimicrobial fragment from its toxicity quencher. In turn, the positively charged antimicrobial fragment interacts with the negatively charged components of the *C. albicans* or *P. gingivalis* cellular membrane, causing interference in electrostatic structure and ultimately, cell death. Alternatively, under healthy conditions, the protease biomarkers are not expressed and the zwitterionic prodrug remains intact, causing no destruction to the cellular membrane.

**Figure 1:**
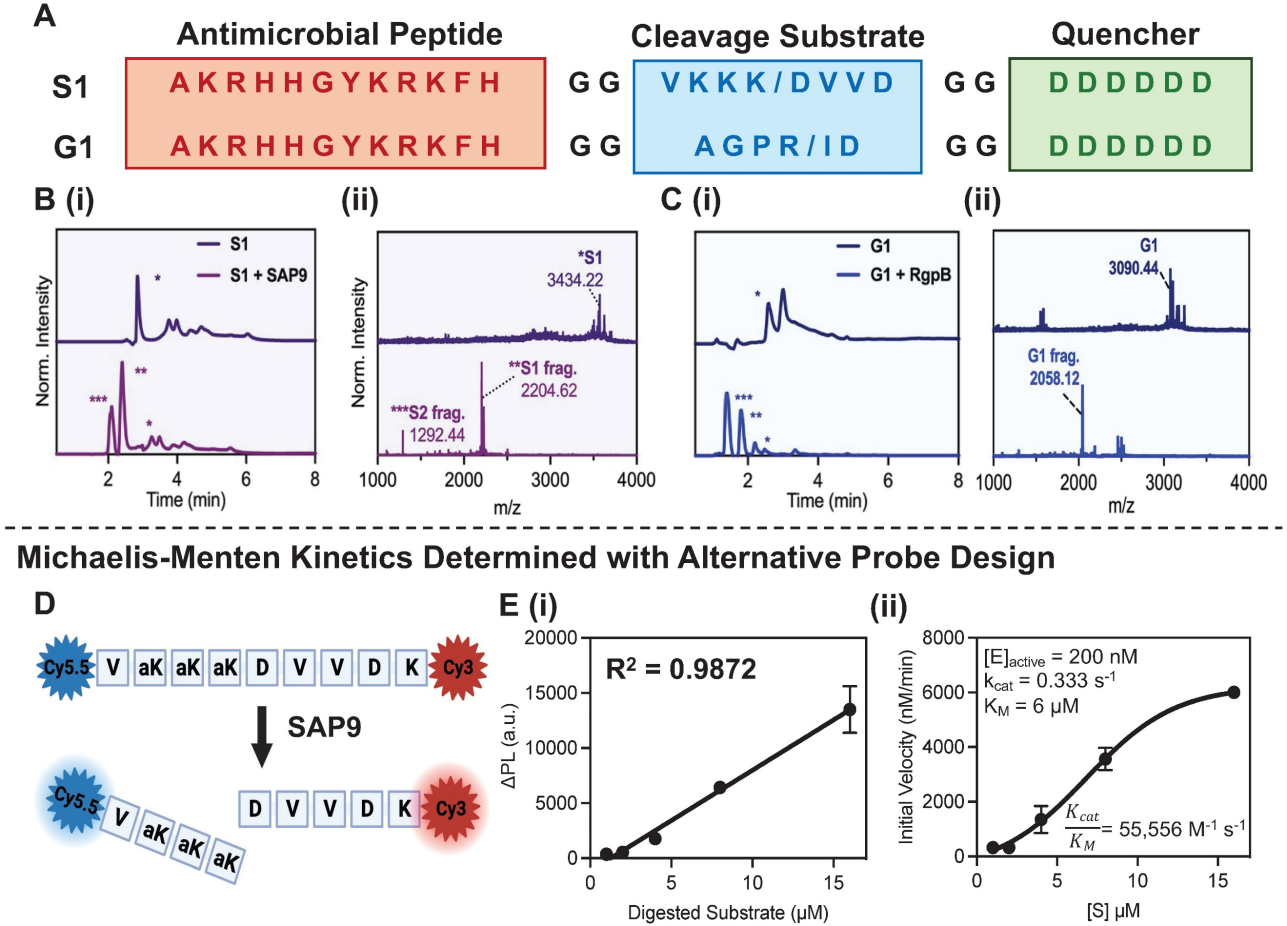
** Activatable antimicrobial peptide design and cleavage characterization. (A)** *C. albicans* and *P. gingivalis* cleavable prodrugs, S1 (top) and G1 (bottom), respectively demonstrating the three main components: **(1)** A positively charged antimicrobial peptide, P-113 (red), **(2)** a cleavable peptide substrate (blue) and **(3)** a negatively charged toxicity quencher (green). Enzymes expressed by *C. albicans* (SAP9) can cleave the S1 prodrug after the third lysine (K/D) in the cleavage substrate, thus resulting in the release of the antimicrobial fragment and subsequent therapeutic activity. Enzymes expressed by *P. gingivalis* (RgpB) can cleave the G1 prodrug after the arginine residue in the corresponding cleavage substrate (R/I)and render the same effect.** (B) (i)** HPLC (220 nm wavelength monitoring) and **(ii)** MALDI-TOF spectra of S1 prodrug intact (top) and cleaved (bottom), i.e., after 3 hours of incubation with 300 nM of SAP9) show a divide in the spectra with corresponding peaks at fragment mass. * S1 = S1 intact, ** S1 = S1 antimicrobial fragment, *** S1 = S1 toxicity blocker fragment. **(C) (i)** HPLC (220 nm wavelength monitoring) and **(ii)** MALDI-TOF spectra of G1 prodrug intact (top) and cleaved (bottom, i.e., after 3 hours of incubation with 200 nM of RgpB) showing a divide in the spectra with corresponding peaks at fragment mass. * G1 = G1 intact, ** G1 = G1 antimicrobial fragment, *** G1 = G1 toxicity blocker fragment. The missing peak in panel C(ii), corresponding to the toxicity fragment of G1, was later identified via ESI-MS (**Supplemental Figure S7**).** (D)** Structure of SAP9 cleavable heterodimer FRET probe with NHS-amine reacted cyanine dyes conjugated on either terminal to create strong fluorescence quenching when intact for estimates of Michalis-Menten parameters.** (E) (i)** Standard curve of linear increase in change in fluorescence signal as a result of enzyme incubation over time with respect to substrate concentration. **(ii)** Michaelis-Menten master curve describing the rate of reaction with an increase in substrate concentration to determine k_cat_/ K_M_, the second-order rate constant reaction rate of the enzyme-substrate complex to product. Here, a higher ratio suggests a higher rate of conversion. Inset parameters present: [E]_active_, the active enzyme concentration used, 200 nM. Catalytic constant, k_cat_, the number of probe molecules converted by enzyme per second. The Michaelis-Menten constant, K_M_, an inverse measurement of affinity. Error bars in panel E represent standard deviation of replicate experiments.

**Design of prodrugs specific to SAP9 or RgpB.** Two activable prodrugs unique to *C. albicans* or *P. gingivalis* were designed with three functional parts (**Figure 1A**). The first part (**Scheme 1** red) is the antimicrobial peptide (**AMP**; P-113; **AKRHHGYKRKFH; Table 1**). This 12-amino-acid derivative of Histatin-5 has antifungal activity comparable to that of the parent molecule with fewer sites prone to proteolytic degradation 41. P-113's electrostatic interference (net charge: +5) causes pore-like breakdowns of the cellular wall, leakage of intracellular contents, and ultimately, cell death (**Scheme 1**) 42. This process is rapid and broad spectrum 43, 44. Alike work suggests that P-113 inhibits biofilm formation and kills periodontal bacteria, such as *P. gingivalis,* at a low concentration 45, 46. Thus, we hypothesized that P-113 would perform as a joint antifungal and antibacterial treatment 41, 47.

The second part (**Scheme 1** green) blocks antimicrobial activity before cleavage via a poly-aspartic acid sequence (DDDDDD). These negative charges balance the positive charge of P-113 creating a zwitterionic peptide that is non-toxic until proteolytic secretion separates the therapy from the quencher. A neutral charge was selected to avoid repulsion from the negatively charged fungal/ bacterial membrane. Similarly, any initial positive charge could exert premature antimicrobial activity at high concentrations.

The third part (**Scheme 1** blue) is the tunable cleavage sequence and makes the prodrugs selective to *C. albicans* or *P. gingivalis*. *C. albicans* releases a portfolio of proteases, but we chose SAP9 because it is representative of the *Candida* genus 34. SAP9 cleaves primarily after basic amino acids such as lysine^15^—particularly a triple lysine residue. We thus chose the substrate VKKK/DVVD 31. For *P. gingivalis*, we used arginine-gingipain B (RgpB)—an endopeptidase that can cleave peptides on the C-terminal of arginine 18. Particularly, RgpB cleaves between arginine and isoleucine (Arg/Ile), and AGPR/ID is a substrate with a high affinity for RgpB 18. Hence, the specific substrates for either SAP9 or RgpB were incorporated into the peptide design. Di-glycine spacers (GG) were placed between the three functional parts to provide flexibility. We defined the *C. albicans* prodrug as S1 and the *P. gingivalis* prodrug as G1; see Table 1 for full sequences and **Supplemental Figure S1** for full structures.

**Probe activation, kinetics, and cytotoxicity.** P-113, S1, and G1 were synthesized through standard solid-phase Fmoc synthesis, purified using high-performance liquid chromatography (HPLC), and characterized using matrix-assisted laser desorption ionization-time-of-flight (MALDI-TOF) mass spectrometry (see Methods). The cleavage of S1 by SAP9 (300 nM) and G1 by RgpB (200 nM) was first investigated *in vitro* using recombinant proteases via a 3-hour incubation at 37 °C in activity buffer as recommended by previous work (9.5 mM MES, 2.7 mM KCl, 140 mM NaCl, pH 5.5 for SAP9 and 0.2 M Tris HCl pH 7.6, 5 mM CaCl_2_, 100 mM NaCl and 40 mM TCEP for RgpB) 31, 35. MALDI-TOF showed obvious mass peaks corresponding to the cleaved fragments (**Figure 1B-C**). As a control, P-113 was incubated with the proteases, and ESI mass spectrometry showed no degradation (**Supplemental Figure S2**).

Next, the peptides' structure was simulated using PEP-FOLD to illustrate the primary and secondary structures (**Supplemental Figure S3**). P-113 has been shown to coil in buffer, with its unique structure mediating antimicrobial activity 41. Interestingly, the coiled structure of P-113 is maintained after incorporating the sequence into the prodrugs. Thus, once cleaved via the accessible cleavage site, the antimicrobial fragment is expected to exert antimicrobial activity against the microbes.

To evaluate the cleavage efficiency of SAP9, a fluorescent analogue of S1 was synthesized (**Figure 1D, Supplemental Figure S4**) with cyanine dyes conjugated to either terminus to produce FRET quenching. Once cleaved by SAP9, the dyes separate, causing an increased fluorescent signal. The probe was incubated with recombinant SAP9 (200 nM) at 37 °C and fluorescence was monitored longitudinally (**Supplemental Figure S5**) to calculate the k_cat_/K_M_ value as ~55,000 M^-1^ s^-1^ (**Figure 1E**) 48. The cleavage efficiency of RgpB was measured similarly (**Supplemental Figure S6**) and a slightly higher k_cat_/K_M_ value was calculated at ~71,000 M^-1^ s^-1^ 49, 50. The 36% higher k_cat_/K_M_ ratio for RgpB suggests that RgpB works better with less substrate, thus enabling it to cleave the peptide more readily.

**Figure 2:**
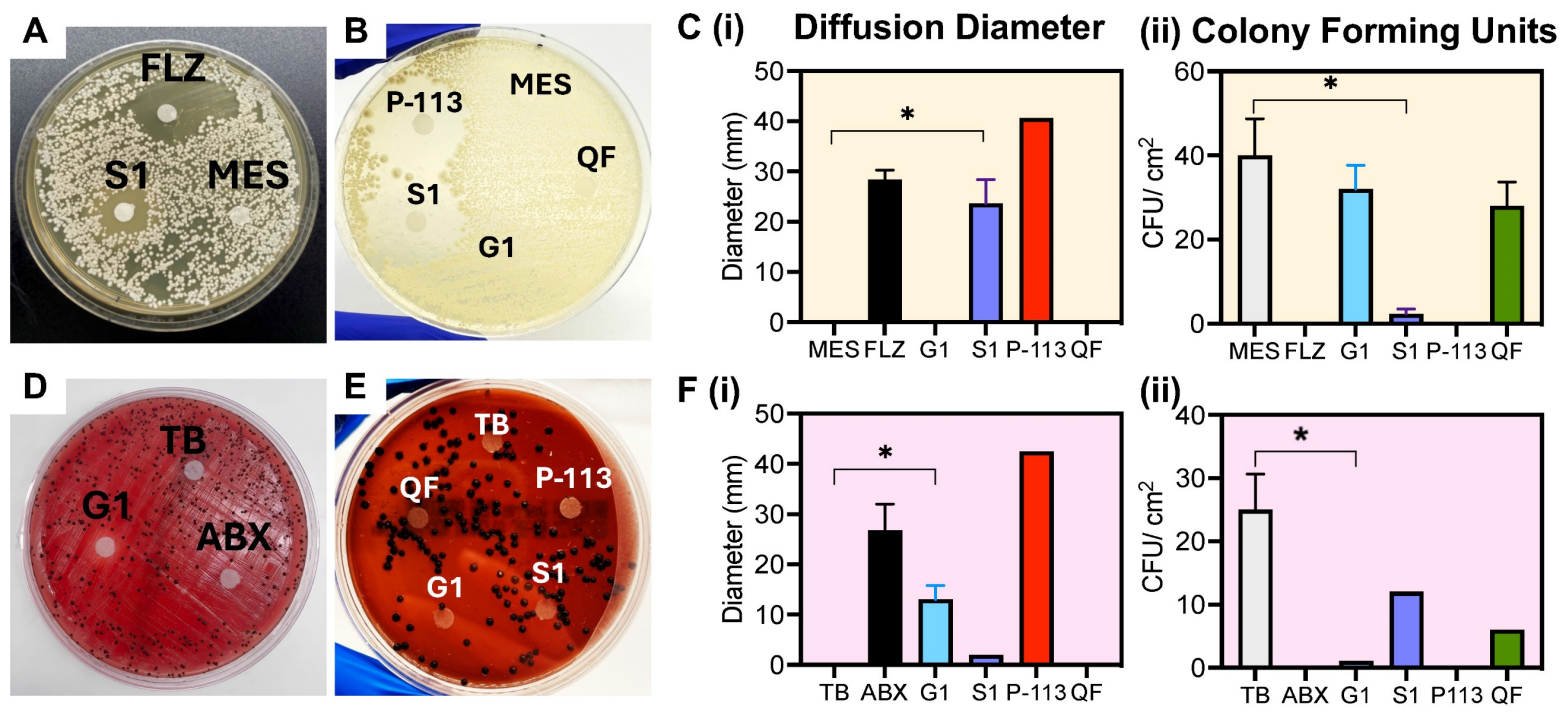
** Microorganism growth and disk diffusion test. (A)** *C. albicans* disk diffusion test after five-day incubation at 30 °C. The disks were treated with 1 mM of: MES buffer (**MES**; negative control), *C. albicans* prodrug (**S1**), fluconazole (**FLZ**; positive control), **(B)** P-113 peptide (**P-113**), toxicity quencher fragment (**QF**) and *P. gingivalis* prodrug (**G1**). Obvious growth inhibition occurs with the experimental (**S1**) and positive controls (**FLZ**, **P-113**). No growth inhibition is observed for the mismatched prodrug (**G1**), buffer, or quencher fragment, as shown by colonies forming around and beneath the treated filter. **(C) (i)** Quantitative diameter measurement and **(C) (ii)** colony forming units surrounding the disk per 1 cm^2^ for disks in panels A and B. **(D)** *P. gingivalis* disk diffusion test after five-day incubation at 37 °C. The disks were treated with 1 mM of: Tris buffer (**TB**; negative control), *P. gingivalis* prodrug (**G1**), penicillin-streptomycin antibiotic (**ABX**; positive control), **(E)** P-113 peptide (**P-113**), toxicity quencher fragment (**QF**) and *C. albicans* prodrug (**S1**). Colony growth is inhibited with the experimental (**G1**) and positive controls (**ABX**, **P-113**). No growth inhibition is observed for the mismatched prodrug (**S1**), buffer, or quencher fragment. **(F) (i)** Quantitative diameter measurement and **(F) (ii)** colony forming units surrounding the disk per 1 cm^2^ for disks in panels D and E. Significance is determined when α < 0.05. Error bars in panels C and F represent the standard deviation of replicate plates.

To verify the prodrugs' antimicrobial activity and proteolytic selectivity, we first grew *C. albicans* and *P. gingivalis* from a lyophilized stock (see Methods, **Supplemental Figure S8**). The presence of secreted aspartyl protease was confirmed via a commercially available ELISA kit as shown in **Supplemental Figure S9**. RgpB secretion was confirmed similarly in previous work 18. Next, a Kirby-Bauer disk diffusion assay was conducted to evaluate initial sensitivity of the microorganisms to the prodrugs. First, the antimicrobial activity of P-113 against the microorganisms was confirmed 51. For *C. albicans*, a clear diameter was formed by addition of S1, showing inhibited growth of the fungus to approximately 70% of the positive control treatment (FLZ; fluconazole) (**Figure 2A-B**). Alternatively, the negative control (MES), mismatched prodrug (G1) and isolated toxicity quencher fragment (QF), all showed colony growth around the disk, demonstrating a lack of toxicity (**Figure 2C**). Similarly, G1 created a 10-mm diffusion diameter on the *P. gingivalis* plate, nearly half that of the antibiotic treatment (**Figure 2D-F**). In comparison, neither the negative control nor the mismatched prodrug showed measurable diffusion of* P. gingivalis* colonies, with rich colony formation near the treatment area. These disk diffusion tests confirm that the prodrug can selectively inhibit the growth of the respective microorganism.

Viability assays were used next to quantitate cleavage-dependent toxicity against *C. albicans* and *P. gingivalis* as well as two control cell lines: *Fusobacterium nucleatum* and human embryonic kidney cells, HEK293T (**Table 2**).* F. nucleatum* is an interesting negative control because it is also present in the gingival sulcus but is commensal and not known to produce either secreted aspartyl proteases or gingipains 18. Mammalian cells were unlikely to be targeted by the prodrugs due to differences in their lipid bilayer membrane structure that is electrically neutral 52.

**Figure 3:**
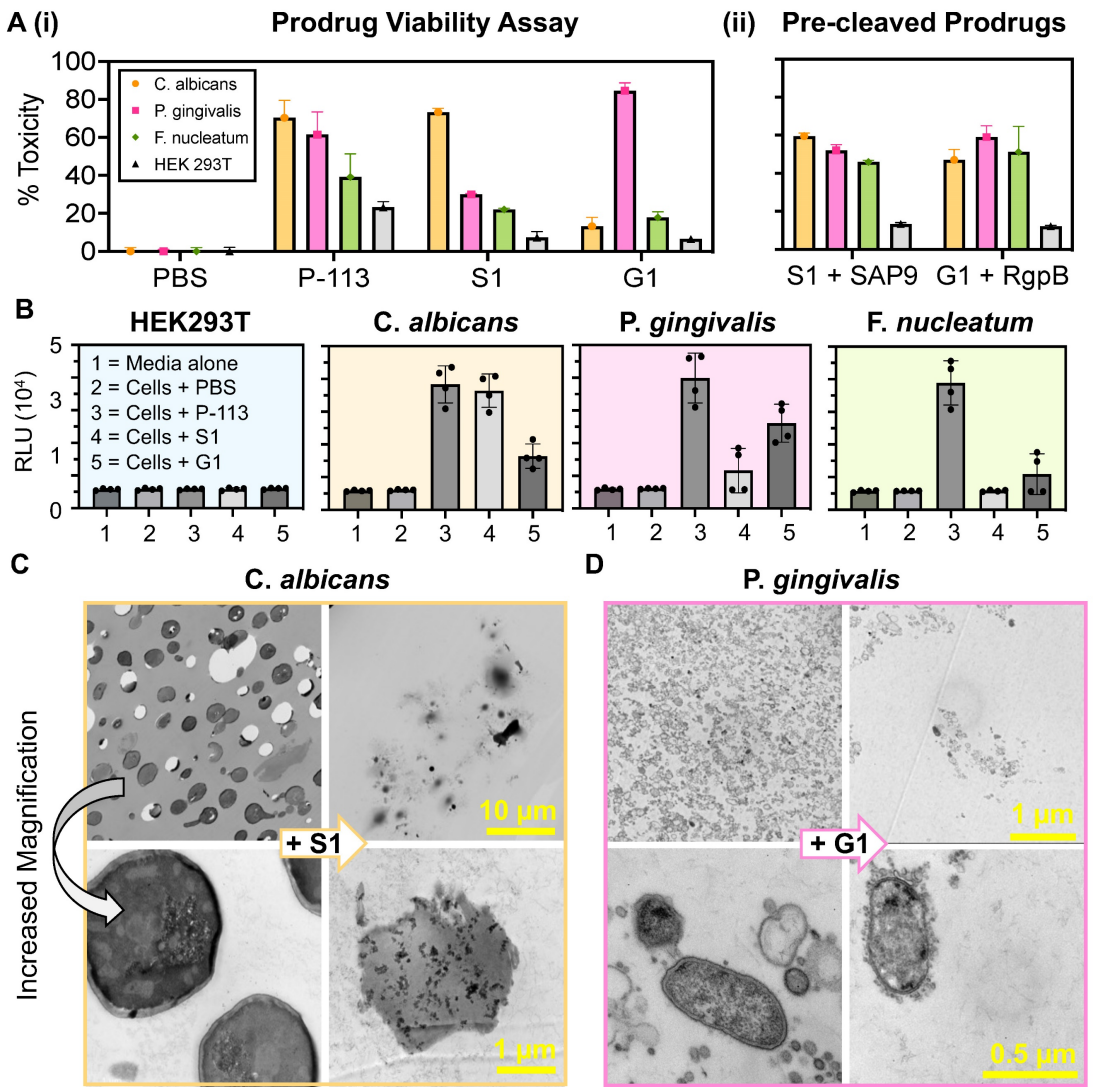
** Influence of prodrugs on oral microorganisms and healthy mammalian cells. (A) (i)** XTT viability assay at 10 µM treatments of: PBS (negative control), P-113 (positive control), S1 (*C. albicans* prodrug), and G1 (*P. gingivalis* prodrug) in four cell lines: *C. albicans*, *P. gingivalis*, *F. nucleatum*, and HEK 293T. Percent toxicity shows that P-113 has an increased effect on oral microorganisms. Conversely, S1 has a negligible effect on all cell lines apart from that which secrete SAP9 (*C. albicans*). Similarly, G1 only exerts a toxic effect greater than 50% against *P. gingivalis*. **(ii)** Viability assay for S1 and G1 cleaved by 300 nM or 200 nM, respectively, of recombinant proteases *in vitro* prior to cell treatment. Here, a return in toxicity similar to that exerted by P-113 is prevalent in all three oral microorganisms. Still, a decreased toxicity is exerted upon HEK293T. **(B)** The effect of prodrugs on ROS generation. Luminescence data show H_2_O_2_ ROS generation in HEK293T, *C. albicans*, *P. gingivalis*, and *F. nucleatum* treated with the prodrugs. The relative luminescence (RLU) obtained from the cells treated with P-113 served as a positive control and the cells without peptide (Cells + PBS) served as the negative control. Error bars in panels A and B represent standard deviation from replicate experiments. **(C)** and** (D)** Transmission electron microscopy (TEM) of 70 nm thin sections of plastic embedded samples to visualize cellular organization. **(C)** Scale bar presents 10 µm (top) and 1 µm (bottom). *C. albicans* (**C.a.**) cells alone, showing dense cellular population at low magnification and rigid, intact cell membrane at high magnification (left). *C. albicans* cells with 40 µM S1 treatment after incubation for 3 hours at 37 °C (**+ S1**) demonstrating consequential decreased cell density and cell rupturing (right). **(D)** Scale bar presents 1 µm (top) and 0.5 µm (bottom). *P. gingivalis* (**P.g.**) cells alone showing full growth and clear in-tact outer membrane (left). *P. gingivalis cells* with 40 µM G1 treatment after incubation for 3 hours at 37 °C (**+ G1**) demonstrating overall cell density decrease and cell-membrane rupture and leakage (right). Different magnifications are shown in Supplemental Information.

Here, the viability of the cell lines outlined in **Table 2** was assessed with 10 µM treatment of PBS, P-113, S1, and G1 (**Figure 3A(i)**) using an XTT viability assay. The incubation time was optimized to 3 hours to allow for successful cleavage and therapeutic release. Excess time (> 6 hours) led to prodrug degradation and eventually (> 9 hours) cellular regrowth as shown by a decrease in percent toxicity. Similarly, insufficient time (< 3 hours) resulted in a decreased toxic effect, suggesting a lower product turnover rate of the prodrugs (**Supplemental Figure S10**). These findings are consistent for both S1 and G1.

The minimum inhibitory concentration (MIC) was an average of 9.5 µM for P-113 against the three oral microorganisms and nearly 100 times that for HEK293T 53. It is worth noting that HEK293T cells were also significantly less affected by P-113, with a toxicity rate that never exceeded 50%, even at 100 µM treatment. This confirms the charge dependence of the P-113 cytotoxic mechanism and corroborates with previous work reporting a MIC of 10 µM for P-113 against *C. albicans* 54.

S1 and G1 were toxic to* C. albicans* (70%) and *P. gingivalis* (82%) at 10 µM. The slight increase in toxicity of G1 in bacteria can be explained by the 36% higher k_cat_/K_M_ value of RgpB. Alternatively, the toxicity of S1 and G1 against *F. nucleatum* never exceeded 50% and showed low toxicity (15%) at 10 µM. The prodrugs, S1 and G1, also showed a decreased toxicity by nearly half to HEK293T, compared to P-113, suggesting that the intact zwitterionic structure reduced toxicity to mammalian cells. Confocal microscopy was used to image HEK293T to ensure that the membrane remained intact after treatment by S1 and G1 (**Supplementary Figure S11**). Importantly, when S1 was pre-cleaved *in vitro* by 300 nM recombinant SAP9 and G1 by 200 nM recombinant RgpB, the resulting compounds expressed toxicity towards the three oral microorganisms (**Figure 3A(ii)**). The percent toxicity of S1 against *P. gingivalis* was originally reported at 30% which nearly doubled upon cleavage of S1 by recombinant SAP9. Similarly, the percent toxicity of G1 against *C. albicans* was reported at 13% which then increased to 47% upon cleavage of G1 by recombinant RgpB- a 3.6-fold increase. This, in turn, confirms that once cleaved, the prodrugs, S1 and G1, release the fragment responsible for cell death. Importantly, the toxicity of the cleaved prodrug has near molar equivalency to the P-113 treatment in the target microorganism, suggesting a return of toxicity after cleavage. Full titration curves are in **Supplemental Figure S12-S16,** with statistical analyses (**Supplemental Figure S17**).

To determine whether the prodrugs' killing mechanism was related to reactive oxygen species (ROS) production, an H_2_O_2_ assay was used to measure the levels of hydrogen peroxide within cells after incubation with the prodrugs at 10 μM for 4 hours. The assay uses an H_2_O_2_ substrate that reacts directly with H_2_O_2_ to generate a luciferin precursor 55. It was predicted that exposure of the microorganisms to the prodrug would lead to alterations in their normal physiological processes thus triggering a stress response and inducing the generation of ROS. As shown in **Figure 3B** a six-fold increase in relative luminescence was observed in cells treated with P-113 and the respective prodrug (i.e., S1 against *C. albicans*, and G1 against *P. gingivalis*). Conversely, little luminescence was observed in cells treated with PBS or the mismatched prodrug (i.e., G1 against *C. albicans* and S1 against *P. gingivalis*). This is consistent with previous studies wherein ROS production is credited as the secondary event of the fungicidal mechanism of P-113 56.

It is worth noting that very related proteases may activate the prodrug (e.g., SAP analogues and RgpA). Nevertheless, this antimicrobial data is powerful evidence that the prodrugs are indeed specific to *C. albicans/ P. gingivalis*. A MEROPS analysis (**Supplemental Figure S18**) was conducted to identify the peptidases expressed by the microorganisms that can degrade the toxicity quencher and release premature therapy. Because the peptidases are consistent between *C. albicans/ P. gingivalis*, this thus confirmed that cross-examination is sufficient to prove selectivity to SAP9/ RgpB.

Finally, the target microorganisms were also imaged using transmission electron microscopy (TEM) to visualize the cell morphology. **Figure 3C** presents *C. albicans* cells. On the left, the untreated cells show a rigid and intact membrane in contrast to the left (+ S1) showing decreased cell density and clear destruction of the membranes. This obvious change in morphology is seen at other magnifications (**Supplementary Figure S19**). Similarly, **Figure 3D** shows in-tact *P. gingivalis* cells with the characteristic gram-negative outer membrane (left) versus treated bacteria (+G1) with decreased cell density and cellular leakage characteristic of apoptosis (**Supplementary Figure S20**). Finally, the cells were imaged with mismatched treatment (i.e., G1 against *C. albicans* and S1 against *P. gingivalis*) and showed no obvious apoptosis, further validating that the prodrugs cannot be “turned-on” under these proteolytic conditions (**Supplementary Figure S21**).

**Scheme 2:**
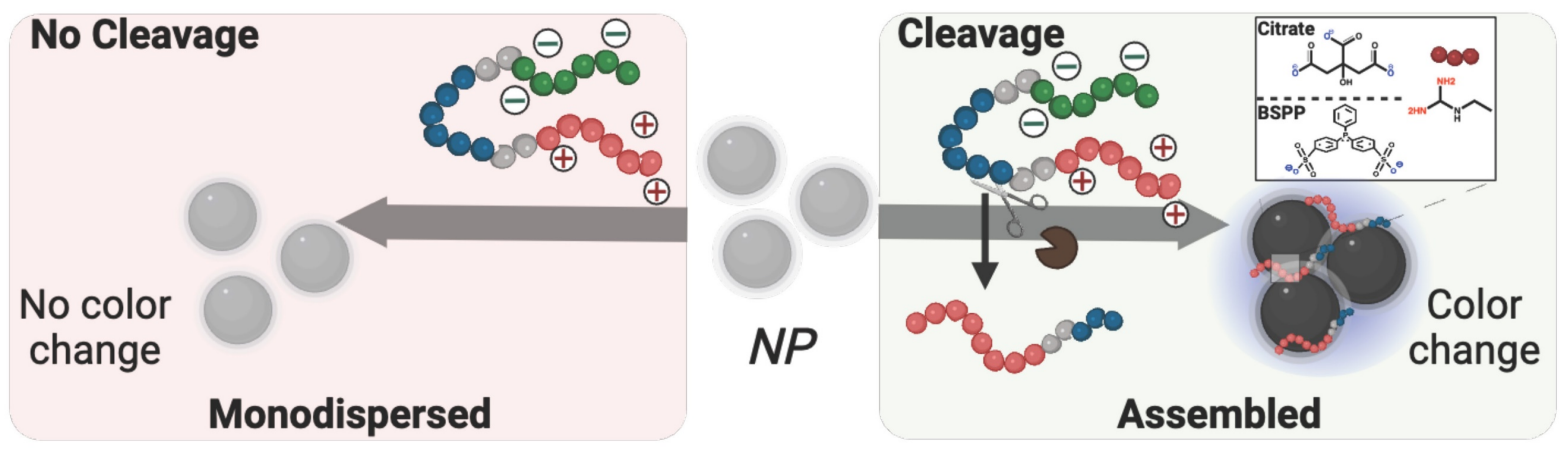
** Descriptive schematic of enzymatic detection with plasmonic nanoparticles.** Color evolution of monodispersed coated-nanoparticles in the presence of intact zwitterionic prodrug (left) vs. the cleavage-induced assembly of the nanoparticles caused by pathogenic enzymatic activity. Citrate-coated gold nanoparticles, (right inset, top) or BSPP-coated silver nanoparticles (right inset, bottom) were used as independent colorimetric systems for comparison. The cleavage of the prodrug results in a color change from ruby red to deep blue for AuNPs and yellow to bright blue for AgNPs.

**Colorimetric detection of pathogens.** Beyond therapy, this system could offer diagnostic utility. Detection of SAP9 and RgpB is important because it is a direct marker of pathogenesis with few convenient detection methods available—especially chairside methods for dental professionals 55. Colorimetric detection via plasmonic particles offers a convenient, point-of-care advantage over conventional or symptomatic diagnoses 38, 56, 57. Previous work reports enzymatic detection via photoluminescence 58, or naked-eye detection of the entire microorganism 59, 60. Herein, we investigated the controlled assembly of gold (AuNPs) and silver (AgNPs) nanoparticles for the colorimetric detection of pathogenic proteases 61. Citrate-capped AuNPs (15 nm diameter) were synthesized 62, and AgNPs (20 nm diameter) were coated with bis(p-sulfonatophenyl)phenylphosphine (BSPP) via a seed-mediated growth procedure 61. We previously demonstrated that the BSPP coating on silver, unlike citrate, can desorb from the AgNPs during positively charged peptide-mediated assembly, thus releasing Ag+ into the solution and permitting spontaneous assembly of the particles 35. Both AuNP-citrate and AgNP-BSPP possess a distinct wavelength of light absorption as well as an associated extinction coefficient. The assembly for the nanoparticle systems is thus promoted via electrostatic interaction between the negatively charged particle surface and the positively charged fragment released after proteolytic cleavage (**Scheme 2**).

**Figure 4:**
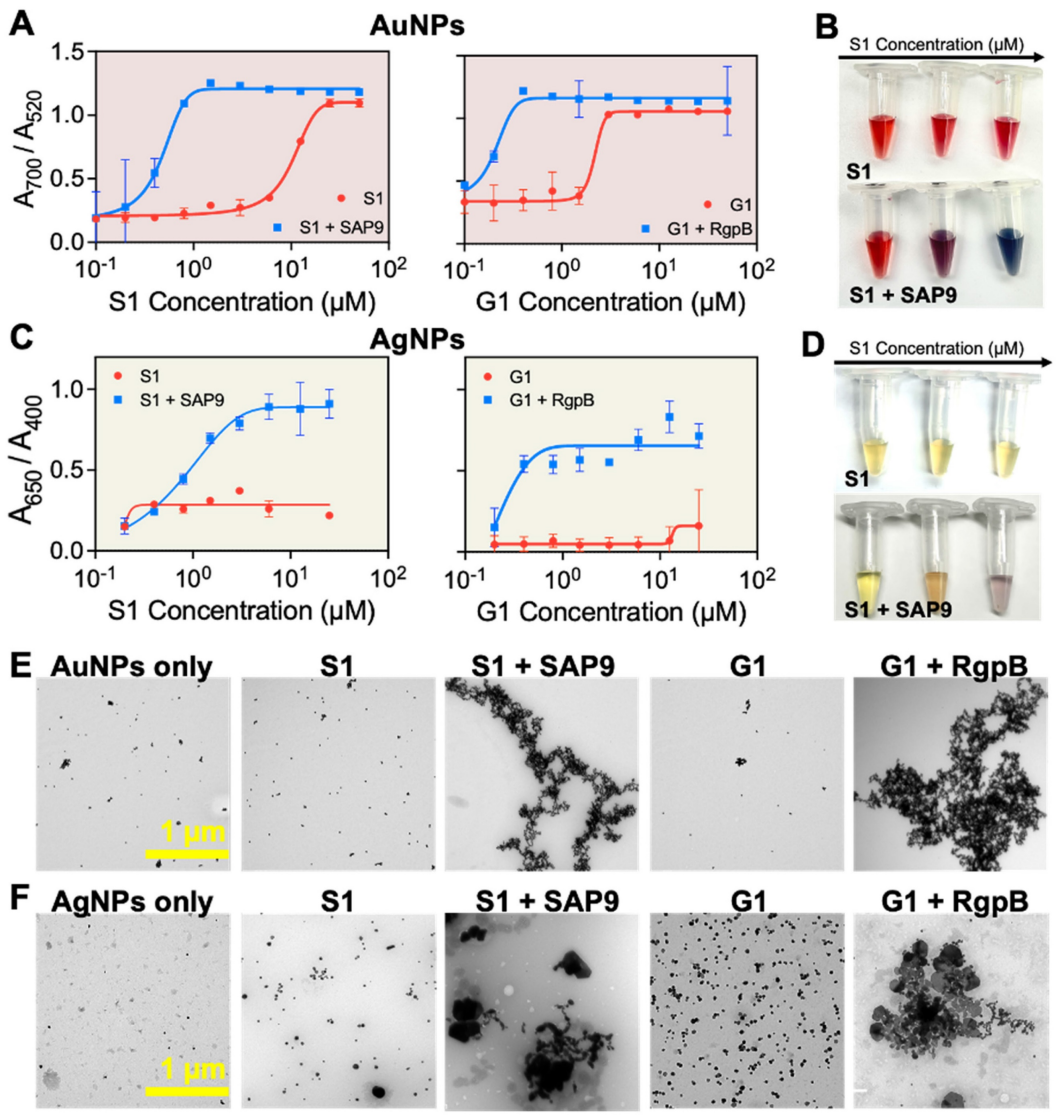
** Protease-induced plasmonic nanoparticle assembly. (A)** Optical absorption of AuNPs when incubated with increased concentrations of S1 (left) and G1 (right) parent (red) and fragmented peptides (blue). **(B)** The color evolution of AuNPs in the presence of intact S1 (control) and cleaved S1 by recombinant SAP9 in increasing concentration from 0.01 to 10 µM wherein high concentration of cleaved peptide results in a transition from ruby red to deep blue, indicative of increased particle assembly caused by isolated positive fragments post-cleavage. **(C)** Optical absorption of AgNPs when incubated with S1 (left) and G1 (right) parent and fragmented peptides. **(D)** The color evolutions of AgNPs in the presence of intact S1 (control) and cleaved S1 by recombinant SAP9 in increasing concentration from 0.01 to 10 µM. Color change from yellow to purple represents increased particle assembly. **(E)** Corresponding TEM images confirming monodispersion or assembly of AuNPs and **(F)** AgNPs when treated with PBS (**NPs only**), intact S1 (**S1**), cleaved S1 (**S1 + SAP9**), intact G1 (**G1**) and, cleaved G1 (**G1 + RgpB**) (left to right). Error bars in panels A and C represent standard deviation from replicate experiments.

First, the AuNP-citrate were mixed with S1 and G1, independently. The zwitterionic design is interesting for controlled assembly of plasmonic nanoparticles because the charge-shielding site effectively quenches the positively charged assembling site prior to proteolytic activity. The colloidal state of the AuNPs-citrate suspension was defined as the ratio of the absorbance: A_700 nm_/A_520 nm_ 39, 40, 63. High and low ratios correspond to assembled or monodispersed NPs, respectively. The maximum ratio was obtained for concentrations of 25 µM and 5 µM of S1 and G1, respectively. However, when S1 and G1 were cleaved by recombinant proteases, the concentration needed to induce the assembly dropped to 1.5 and 0.4 µM for S1 and G1, respectively, showing that the colloidal state is responsive to biomarker activity (**Figure 4A**). Visually, the AuNP-citrate suspensions turned from deep red to blue almost instantly after addition in the presence of the proteases (**Figure 4B**). AgNP-BSPP were used similarly wherein the colloidal state of the suspension was defined as the ratio of the absorbance: A_650 nm_/A_400 nm_ 61. The maximum ratio was obtained at 13 µM for both S1 and G1. Here, the intact prodrugs were unable to aggregate the particles at the highest concentration, expanding the working window for the silver system. However, when S1 and G1 were cleaved with the corresponding protease, the concentration needed to assemble the silver nanoparticles dropped to 6 µM and 0.4 µM, again showing that the colloidal state is responsive to the protease activity (**Figures 4C**). Similarly, visual detection was obvious for AgNPs in a transition from yellow to purple (**Figure 4D**). This was repeated in saliva to demonstrate the applicability of this detection system in complex media. Here, 10% saliva returned a similar trend (**Supplemental Figure S20**). The assembly was further verified through TEM (**Figure 4E-F**) demonstrating the transition from monodispersed to peptide-assembled as a consequence of the cleavage of the prodrugs by the proteases.

**Figure 5:**
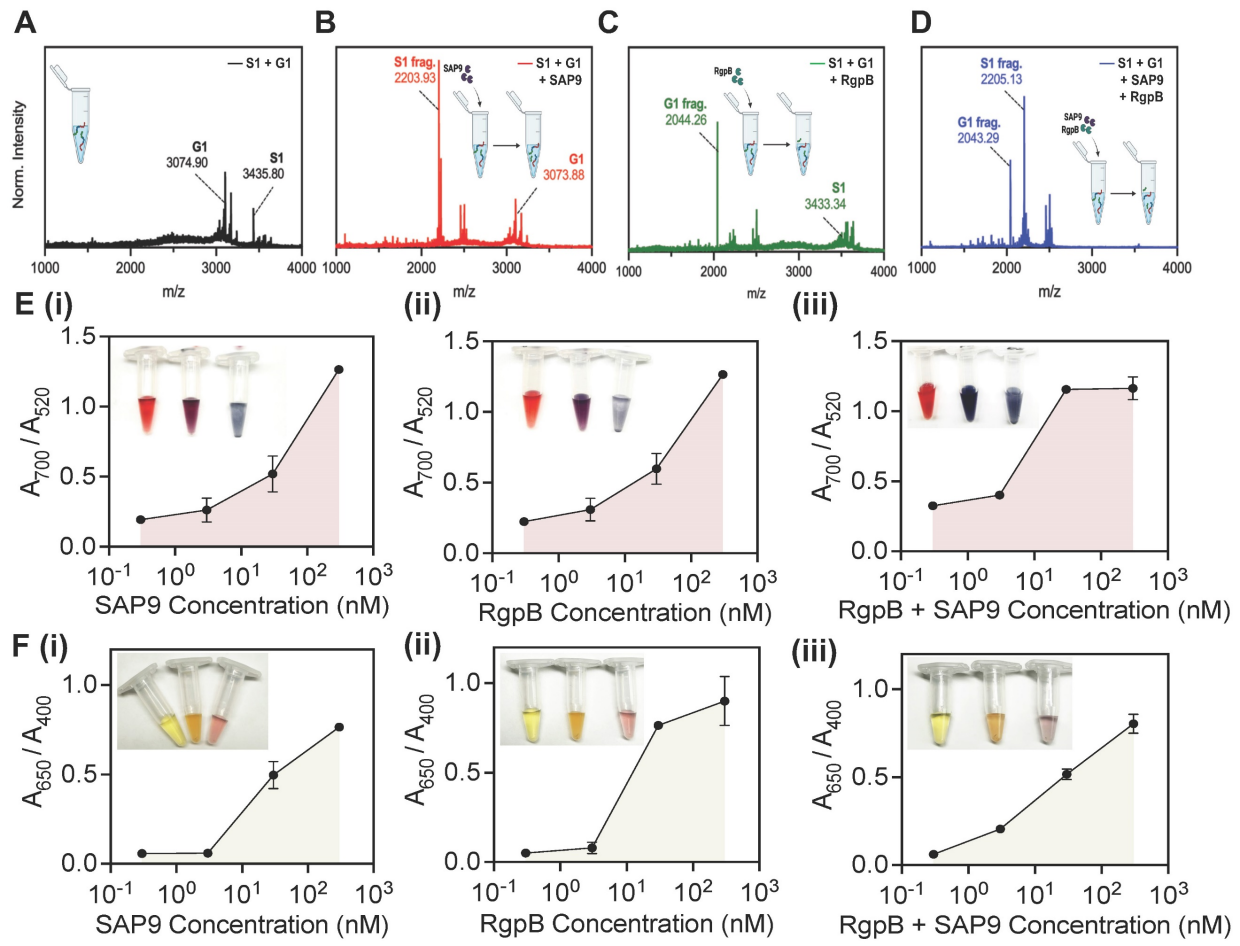
** Combined cleavage and limit of detection.** MALDI-TOF of **(A)** joint incubation of intact S1 and G1 peptides showing successful synthesis and co-incubation. **(B)** S1 and G1 incubated with SAP9 (300 nM, 3 hours, 37 °C), showing S1 converting to S1 fragment (2203 Da), and intact G1, with no peak corresponding to a G1 fragment. **(C)** S1 and G1 incubated with RgpB (300nM, 3 hours, 37 °C), showing S1 remaining intact, and G1 cleaving as confirmed by the G1 fragment peak (2044 Da). **(D)** S1 and G1 incubated with 300 nM SAP9 and RgpB for 3 hours at 37 °C, showing both prodrugs cleaving as presented by only fragment peaks. **(E)** Limit of detection (LOD) for AuNP-citrate and **(F)** AgNP-BSPP detection system of co-incubated S1 and G1 using an increasing concentration of **(i)** SAP9, **(ii)** RgpB, and **(iii)** both, SAP9 and RgpB. LOD study shows a decrease in the limit for both proteases and a detectable limit within the regime of physiologically relevant protease secretion concentrations. Inset presents color evolution at 0 nM, 3 nM, and 300 nM (left to right). Error bars in panels E and F represent standard deviation from replicate experiments.

Next, the peptides were investigated together in solution. The goal was to optimize the system to both *C. albicans* and *P. gingivalis* for a dual sensing mechanism. The peptide-duo was subjected to four conditions: no enzyme, SAP9, RgpB, and an equimolar combination of SAP9 and RgpB. Here, the cleavage of the mixed peptides (**Figure 5A**) was reevaluated using MALDI-TOF. The cleavage confirmation was presented as previously, based on the emerging peaks indicative of the fragment mass. A clear peak for G1 remained present during incubation with SAP9 (**Figure 5B**). Likewise, S1 remained intact when the duo-peptide solution was incubated with RgpB, thus demonstrating the affinity of the proteases to their cleavage substrate (**Figure 5C**). Only during the coincubation with both proteases were both prodrugs cleaved (**Figure 5D**).

The peptides were next subjected to a colorimetric assay with AuNPs at varying concentrations of the enzymes. A limit of detection (LOD) of 10 nM (**Figure E, i**) was obtained for SAP9, which agrees with previously reported protease detection systems 18, 38, 39. The color change was also clear as an increased enzymatic concentration returned a blue suspension. RgpB yielded a similar LOD with the gold system at approximately 10 nM (**Figure E, ii**). Conversely, when both enzymes were incubated in the duo-peptide solution, the LOD was nearly halved as suggested by the more intense color change and enthusiastic assembly of the particles. Here, 3 nM protease treatment resulted in a deep blue color versus the previous purple color (**Figure E, iii**).

The silver system proved more sensitive, with a LOD of approximately 3 nM for SAP9 and RgpB (**Figure F, i-ii**) and returned an obvious color change (yellow to purple). The system was, again, more responsive when both proteases were present (LOD = 0.3 nM) and the color change followed in a more dramatic change from yellow to purple-blue (**Figure F, iii**). The tenfold increase in sensitivity of the silver system is consistent with previous work 35. This data further suggests that due to their ability to produce a stronger plasmon resonance than gold, silver is able to absorb light more readily making it more sensitive to peptide assembly.

Finally, this limit was assessed in saliva (**Supplemental Figure S23**). Here, the LOD increased by a factor of 10 for both enzymes. It is likely that saliva interferes with nanoparticle sensors by suppressing color change signals due to nonspecific protein absorption 64. As such, this increase is expected. Another explanation for this is the negatively charged proteins forming a protein corona around the nanoparticles thus impeding the aggregation 65. It is worth noting that several factors influence the LOD, including the specificity of the detection method, the affinity of the probe for the target protease, the signal amplification strategies employed, and the background noise in the system. Literature cites gingipain concentrations within a nanomolar range in human gingival crevicular fluid samples. Similarly, SAP levels involved in specific signaling pathways or regulatory processes are present in the nanomolar range in candidiasis patients 49, 66-69. Given the kinetic information of the design and the low MIC yielded, the LOD is satisfactory and in line with the physiological concentration of SAP9/RgpB.

## Conclusion

We report a therapeutic and diagnostic scaffold for *C. albicans* and *P. gingivalis* that harnesses the value of antimicrobial peptides for oral health. Although P-113 has been pursued for years due to its antimicrobial properties, the activatable form is a novel approach to the development of antimicrobial therapies. By offering specific release, the prodrug-peptides demonstrated high toxicity (MIC = 6.02 µg/ mL) towards the microorganisms expressing the trigger protease. Alternatively, when subjected to conditions absent of the target, the prodrugs delivered relatively no toxicity and never exceeded 50% cell death even at concentrations as high as 1 mM. Finally, the charge-switchable design was exploited for *in vitro* colorimetric detection using plasmonic nanoparticles, a feature of P-113 that had yet to be explored. Validation of this system demonstrated rapid detection of the relevant toxicity markers of *C. albicans* and *P. gingivalis* at nanomolar enzymatic concentrations. Importantly, this combination theragnostic approach is promising for developing controlled preventative treatment and enhanced diagnoses in the oral microbiome. It is worth noting that increasing the affinity of the proteases to the cleavage substrate can improve the effectiveness of the therapy and limit of detection. We imagine that this prodrug strategy can be extended to any enzyme after identifying the appropriate peptide substrate making a sustainable approach to theranostic devices.

## Experimental Section

**Enzyme digestion.** SAP9 and RgpB cleavage were both performed with 455 µM of peptide. Typically, 50 µL of peptide (1 mM) were mixed with 50 µL of buffer (9.5 mM MES, 2.7 mM KCl, 140 mM NaCl, pH 5.5 for SAP9 and 0.2 M Tris HCl pH 7.6, 5 mM CaCl_2_, 100 mM NaCl and 40 mM TCEP for RgpB). Then, 10 µL of the enzyme at the appropriate concentration was added. An optimized final concentration of 300 nM for SAP9 and 200 nM for RgpB were used. The solution was incubated at 37ºC for 3 hours for RgpB and SAP9 for optimal cleavage.

**Fungal culture.** *In situ* culturing *C. albicans* (ATCC 90028) from lyophilized stock (KWIK-STIK, VWR) was conducted on Sabouraud dextrose agar plates (Sigma-Aldrich) 2 to 5 days in advance of the antifungal assay 70. The plates were then incubated at 30°C and pearl-like colonies were observed, indicative of *C. albicans*. Single colonies were then extracted with sterilized inoculation loops and inoculated in 3 mL yeast extract peptone dextrose (YEPD) medium (2% Bacto^TM^peptone, 2% Dextrose, 1% Yeast extract in H_2_O) using Biological Safety Level 2 (BSL-2) practices and procedures. Note, the broth was autoclaved before use and stored at room temperature. The inoculates were then grown overnight with gentle shaking at 30 °C. The culture was finally diluted 1:100 and the OD600 was measured wherein OD600 = 0.5 a.u. correlates to approximately 1 × 10^6^ cells/ mL in 100 µL RPMI-1640 medium with L-glutamine and 3-(N-morpholino)propane sulfonic acid (MOPS) without sodium bicarbonate, pH 7.0. Next, the 96-well plates were sealed with a sealing membrane to reduce evaporation and prevent cross contamination before being incubated at 37°C to allow biofilms to form overnight (**Supplemental Figure S24**). The media was then carefully discarded, and the biofilms were washed from free cells with 200 µL sterile PBS.

**Bacteria culture.** *P. gingivalis* and *F. nucleatum* colonies were grown on brucella blood agar plates (Sigma) from lyophilized stocks of ATCC 33277 and ATCC 25586, respectively (KWIK-STIK, VWR) 18. Anaerobic atmospheres were generated and maintained using anaerobic bags with oxygen indicators (GasPak EZ Pouch System, BD). Briefly, the plates were streaked with bacteria-loaded swabs and immediately placed in the gas-generating sachets. For *P. gingivalis*, light-colored colonies became visible 4-5 days of incubation at 37°C; these colonies turned black over the following four days due to heme accumulation/metabolism (signature of *P. gingivalis*). *F. nucleatum* colonies were faster growing (2-4 days) and maintained a light color. Single colonies were transferred to enriched tryptic soy broth (eTSB) which was prepared at 500-mL scale by dissolving 15 g tryptic soy broth and 2.5 g yeast extract in 450 mL DI water followed by addition of 2.5 mL hemin (1 mg/mL). The pH was adjusted to 7.4 using 5 M NaOH and water was added to reach 500 mL. The broth was then autoclaved followed by aseptic addition of 5% (w/v) L-cysteine, 10 mL 1% (w/v) dithiothreitol, and 2 mL menadione (0.5 mg/mL) 18.

**Viability assay.** The percent toxicity was calculated using an XTT (2,3-bis-(2-methoxy-4-nitro-5-sulfophenyl)-2H-tetrazolium-5-carboxanilide) colorimetric assay (Biotium) wherein viable cells form an orange formazan product upon reduction. Here, the oral microorganism cells were grown under appropriate conditions until the desired growth phase of 1 × 10^6^ cells/ mL in a 96-well plate. Control wells with medium only served as the blank control and wells with cells treated with fluconazole or penicillin-streptomycin agent served as the positive control. Finally, an increasing concentration of the various peptides were used to treat the cells in sextuplet replicate measurements. The plates were incubated at 37 °C for 3 hours allowing the antifungal agents to exert their effects. XTT solution was then added to each well as recommended by the provider. The plates were then incubated in the dark at the appropriate temperature for 3 hours. The absorbance of the formazan dye was measured at a wavelength of 490 nm using a microplate reader. Here, the absorbance is directly proportional to the number of viable cells present in each well. Thus, higher absorbance indicates higher cell viability. The readings were then normalized by subtracting the average absorbance of the blank control wells. Finally, the percentage of cell viability for each treatment group was calculated by comparing the absorbance of treated wells to the control wells. The MIC was also determined after subtraction of the blank control consisting of growth medium only. Here, readings below 0.01 optical density units (i.e., no visible growth) indicated growth inhibition. Independent MIC determinations with the same isolate and compound were usually identical or within a twofold range. The average was collected along sextuplet measurements collected on two independent plates.

**AuNP synthesis.** Citrate-stabilized AuNPs were synthesized at 15 nm using the Turkevich method 62. Briefly, 45 mg of HAuCl_4_·3H_2_O was dissolved in 300 mL of MQ water while subjected to generous stirring (600 rpm) and boiling conditions at 120 ºC via oil bath. Next, 150 mg of sodium citrate (dissolved in 5 mL of MQ water) was rapidly injected, and the reaction was left under boiling conditions for 20 minutes. The color of solution changed from purple to gray and finally dark red. The resulting product was cooled and stored at room temperature for the future use. The optical density of the final product was 1.45 (concentration ~ 3.6 nM, ɛ_520_ = 4.0 × 108 M^-1^cm^-1^). Notably, the flask used was cleaned thoroughly with Agua regia and distilled water (three times) prior to synthesis.

**AuNP detection.** 100 µL of 15 nm AuNPs-citrate (OD = 1) were mixed with varying concentration of the peptides in buffer, allowing the color of the suspension to turn from bright ruby-red to purple, and ultimately, deep blue.

**AgNP synthesis.** BSPP-coated AgNPs (AgNPs-BSPP) were synthesized using a two-step procedure 61. First, silver seeds were produced by the reduction of silver nitrate (0.1 mM, 6 mL) with sodium borohydride (0.1 M, 60 μL) in a glass vial under magnetic stirring for 16 h. The seeds were then grown into nanoparticles by adding sodium ascorbate (15 mM, 400 μL), BSPP (5 mM, 200 μL), dropwise silver nitrate (1 mM, 4 mL), and then BSPP again (5 mM, 400 μL), and the resulting solution was stirred for 48 h at room temperature.

**AgNP detection.** 100 µL of 20 nm AgNPs-BSPP (OD = 1) were mixed with increasing concentrations of the peptides in aqueous solution, allowing the color of the suspension to turn from bright yellow to orange, red and ultimately, bright blue.

## Supplementary Material

Supplementary materials, experimental methods, instrumentation, synthesis characterization, microscopy magnification variation, and expanded toxicity assay titrations.

## Figures and Tables

**Table 1:** ** Key peptides.** Peptide P-113 is an antimicrobial peptide that serves as a positive control. It is integrated into activatable peptides S1 and G1.

Name	Sequence	Mass (Da)	Charge
**P-113**	Ac-**AKRHHGYKRKFH**	1604.8891	+5
***C. albicans* prodrug (S1)**	Ac-**AKRHHGYKRKFH**GGVKKK/DVVDGGDDDDDD	3434.6778	0
***P. gingivalis* prodrug (G1)**	**AKRHHGYKRKFH**GGAGPR/IDGGDDDDDD	3090.4474	0

**Table 2:** Key microorganisms investigated.

Microorganism	Target Protease	Features
***C. albicans***	SAP9	S1 peptide target
***P. gingivalis***	RgpB	G1 peptide target
***F. nucleatum***	-	Cleavage negative control
**HEK 293T**	-	Toxicity negative control
