# Supplementary material for: Activatable prodrug for controlled release of an antimicrobial peptide via the proteases overexpressed in Candida albicans and Porphyromonas gingivalis
Source: Theranostics. 2024 Feb 17;14(4):1781–93. doi: 10.7150/thno.91165 (PMC10879876; doi:10.7150/thno.91165)
Supplement: Supplementary file 1 — Supplementary materials, experimental methods, instrumentation, synthesis characterization, microscopy magnification variation, and expanded toxicity assay titrations. [file thnov14p1781s1.pdf]

SUPPORTING INFORMATION

Activatable prodrug for controlled release of an antimicrobial peptide via the proteases overexpressed in *Candida albicans* and *Porphyromonas gingivalis*

Lubna Amer,[a] Maurice Retout,[b] and Jesse V. Jokerst[a,b,c*]

[a] Program in Materials Science and Engineering, University of California, San Diego, La Jolla, CA 92093, United States

[b] Department of NanoEngineering, University of California, San Diego, La Jolla, CA 92093, United States

[c] Department of Radiology, University of California, San Diego, La Jolla, CA 92093, United States

***Corresponding author's email:** jjokerst@ucsd.edu (J.V.J.)

Table of Contents

I. Materials ____ 3

I. Experimental Procedures ____ 3

II. Supplementary Data ____ 7

Figure S1. Structure of P-113 and SAP9 and RgpB- cleavable prodrugs, S1 and G1, respectively (top to bottom). ____ 7

Figure S2. P-113 characterization. ____ 7

Figure S3. Simulated secondary structure of prodrugs. ____ 8

Figure S4. SAP9 cleavable substrate FRET probe characterization. ____ 9

Figure S5. SAP9 cleavable substrate FRET probe fluorescence. ____ 10

Figure S6. RgpB cleavable substrate FRET probe characterization. ____ 11

Figure S7. ESI-MS characterization of purified fragment corresponding to G1 toxicity blocker. ____ 12

Figure S8. Microorganism growth for disk diffusion test. ____ 13

Figure S9. ELISA detection of SAP in *C. albicans* culture. ____ 14

Figure S10. Time dependent toxicity assessment for S1 and G1. ____ 15

Figure S11. Confocal microscopy of mammalian cells. ____ 16

Figure S12. Toxicity assessment for varying concentrations of P-113, positive control, treatment along four cell lines. ____ 17

Figure S13. Toxicity assessment for varying concentrations of S1, *C. albicans* prodrug, treatment, along four cell lines. ____ 18

Figure S14. Toxicity assessment for varying concentrations of G1, *P. gingivalis* prodrug, treatment, along four cell lines. ____ 19

Figure S15. Toxicity assessment for varying concentrations of S1 + SAP9, *C. albicans* prodrug pre-cleaved by 200 nM recombinant protease in vitro, treatment, along four cell lines. ____ 20

Figure S16. Toxicity assessment for varying concentrations of G1 + RgpB, *P. gingivalis* prodrug pre-cleaved by 200 nM recombinant protease in vitro, treatment, along four cell lines. ____ 21

Figure S17. Statistical analysis to highlight the antimicrobial specificity of protease-activated prodrugs. ____ 22

Figure S18. MEROPS Analysis. ____ 23

Figure S19. *C. albicans* TEM images of cellular morphology at increased magnification. ____ 24

Figure S20. *P. gingivalis* TEM images of cellular morphology at increased magnification. ____ 25

Figure S21. Intact prodrug effects on membrane integrity. ____ 26

Figure S22. Protease-induced plasmonic nanoparticle assembly in saliva. ____ 27

Figure S23. Limit of detection of plasmonic nanoparticle assembly in saliva. ____ 28

Figure S24. *C. albicans* growth transmission microscopy. ____ 29

III. Supplementary References ____ 30

IV. Author Contributions ____ 30

I. Materials

Fmoc-protected L-amino acids, hexafluorophosphate benzotriazole tetramethyl uranium (HBTU), and Fmoc-Rink amide MBHA resin (0.67 mmol/g, 100-150 mesh) were purchased from AAPPTec, LLC (Louisville, KY). Organic solvents including N,N-dimethylformamide (DMF, sequencing grade), acetonitrile (ACN, HPLC grade), ethyl ether (certified ACS), methylene chloride (DCM, certified ACS), and dimethyl sulfoxide (DMSO, certified ACS) were from Fisher Scientific International, Inc. (Hampton, NH). Ultrapure water (18 MΩ.cm) was obtained from a Milli-Q Academic water purification system (Millipore Corp., Billerica, MA).

Cy5.5-NHS and Cy3-NHS were purchased from Lumiprobe, Inc. (Maryland, USA). Dimethyl sulfoxide, leupeptin, phosphate-buffered saline, tris base/HCl, dithiothreitol, triethylamine, L-cysteine, menadione, yeast extract, tryptic soy broth, dextrose, and hemin were purchased from Sigma-Aldrich (Missouri, USA). Acetonitrile, trifluoroacetic acid, RPMI-1640 medium, 3-(N-morpholino)propane sulfonic acid, Bacto^{TM}peptone, and sodium hydroxide were purchased from Fisher Scientific (Massachusetts, USA). Recombinant SAP9 and SAP ELISA Kit were purchased from MyBioSource (San Diego, CA). Recombinant RgpB was a kind gift from Professor Anthony O'Donoghue (UC San Diego Skaggs School of Pharmacy and Pharmaceutical Sciences, San Diego, CA).

C. albicans (ATCC 90028), *P. gingivalis* (ATCC 33277) and *F. nucleatum* (ATCC 25586) were all purchased as lyophilized stock (KWIK-STIK, VWR, Radnor, PA). Agar plates were also purchased from Sigma-Aldrich (St. Louis, MO). XTT Cell Viability Assay Kit was purchased from Biotium (Fremont, CA).

Citrate-capped silver nanoparticles (OD = 1) were purchased from Nanocomposix (San Diego, CA). Gold(III) chloride hydrate (HAuCl4·3H2O, ≥99.9%), sodium citrate tribasic dihydrate (>99%), Trizma® base (>99.9%), Trizma® hydrochloride (>90%), DL-dithiothreitol (DTT, >99%), were purchased from Tokyo Chemical Industry Co., Ltd (TCI). Sodium chloride (NaCl, certified ACS), urea (certified ACS), and hydrochloric acid (certified ACS) were purchased from Fisher Chemical (Waltham, MA).

I. Experimental Procedures

Peptide synthesis. Peptides were synthesized as prepared in previous work [1]. Briefly, an automated Eclipse™ peptide synthesizer (AAPPTec, Louisville, KY) was utilized for standard solid phase Fmoc synthesis on Rink-amide resin (0.55 mmol/g, 200 mg). Amino acids were coupled (C to N) under nitrogen protection with 0.2 M Fmoc-amino acid (5 equiv.) in 3 mL DMF, 0.2 M HBTU (5 equiv.) in 3 mL DMF, 0.4 M DIPEA (7.5 equiv.) in 3 mL DMF, and 20% (v/v) piperidine in 2 x 4 mL DMF for each coupling cycle. The resulting resin and peptide were then transferred to a syringe filter (Torviq Inc.) and washed with three rounds of DMF (5 mL each) and three rounds of DCM (4 mL each). It was then dried under vacuum. For acetylated peptides, the N-terminal was acetylated using the following recipe: 4 mL of DMF, 0.5 mL of Pyridine, and 0.5 mL of acetic anhydride. The solution was then subjected to light stirred for 30 minutes before being purged and washed with DMF and DCM as previously mentioned. The dried peptides were

next cleaved from the resin using a 5 mL cocktail solution that consists of: 83% TFA, 5% H2O, 5% thioanisole, 5% phenol, and 2% µL EDDET. The incubated solution was gently rotated for 2 hours. The resin was then filtered and the filtrate containing the crude peptide was collected and precipitated using cold ethyl ether (20 mL, –20 ºC) and centrifuged three times (8,000 rpm, 3 minutes). Once the supernatant was removed, the precipitated pellets were dried and re-suspended using 10 mL of ACN/H2O mixtures wherein the percentage of ACN was controlled based on the solubility of the peptide.

Peptide purification and characterization. Peptide purification was conducted as done by Retout et al [1]. Once dry, the crude peptides were purified with a Shimadzu LC-40 HPLC system equipped with a LC-40D solvent delivery module, photodiode array detector SPD-M40, and degassing unit DGU-403. An injection of 2 mL was utilized with a Zorbax 300 BS, C18 column (5 µM, 9.4 × 250 mm) using an elution flow rate of 5 mL/ min over a 40-minute gradient from 10% to 95% acetonitrile in water (0.05% TFA). The peptide bond absorbance of 220 nm was monitored closely, and the elution was collected for characterization. Electrospray ionization mass spectrometry (ESI-MS) on the positive ion mode via the Micromass Quattro Ultima mass spectrometer provided by the Molecular MS Facility (MMSF) at UC San Diego was utilized with an MEOH/ H2O mixture (1:1, v/v) and an injection volume of 5 µL. Some compounds were characterized using matrix-assisted laser desorption/ionization-time of flight (MALDI-TOF) mass spectrometer using a linear positive mode. Here, a-Cyano-4-hydroxycinnamic acid (HCCA) was the matrix utilized at a ratio of 1:3 and 2 µL was placed and dried with a heat gun to be analyzed. Duplicates were used as recommended by the MMSF. Fractions containing the pure peptide as confirmed by electrospray ionization mass spectrometry (ESI-MS, positive ion mode) were lyophilized in a FreeZone Plus 2.5 freeze dry system (Labconco Corp., Kansas, MO) and aliquoted and stored in dry conditions at 2 ºC for further use [2].

Michaelis-Menten kinetics. The peptide substrate was resynthesized with acetylated lysine residues and linked to cyanine-NHS ester dyes (Cy5.5 and Cy3) using the amine groups in the form of NH_3^+ and lysine at the N-terminal and the C-terminal, respectively: (V[aK][aK][aK]DVVDK). Acetylated lysine residues were employed to guide the dye to the lysine on the end terminal. Herein, 0.5 mg of the peptide dissolved in 369 µL of anhydrous DMSO with 31 µL of 1% (v/v) triethylamine (2.25 µmol, 1.5 equiv.) Next, Cy5.5 and Cy3 were added to the solution (3.5 µg, 4.50 µmol) and the solution was covered with aluminum foil and left to stir overnight at 300 rpm. The crude reaction was then dried under vacuum centrifugation using a Vacufuge Plus (Eppendorf, Hamburg) at 60 ºC until light-reflecting pellet was formed. The pellet was then resuspended in 25% ACN/ H_2O (v/v) and separated using HPLC.

The heterodimer was diluted in 9.5 mM MES, 2.7 mM KCl, 140 mM NaCl (pH 5.5) buffer to reach a final [S] and distributed in 24 wells within a 96-well plate for duplicate measurements of each concentration. The enzyme ($[E]_0$ = 200 nM with respect to a final 100 µL volume) was then added to each well. The plate was incubated at 37 °C and the fluorescence intensity (684 nm for Cy5.5 and 570 nm for Cy3) was recorded over 12 h with 1 min intervals between each cycle. Measurements were performed in duplicates. The signal values at 30 min readout time were averaged and plotted against substrate concentrations; error bars represent the standard error of the means. The $\Delta PL = PL_{30\ min} - PL_{0\ min}$ was then correlated to product concentration using a

standard curve: $\Delta PL_{Cy5.5}$ vs. [fully-digested FRET probe]. Data were then fitted to the following Michaelis-Menten equation: $v = \frac{V_{max}[S]}{K_{m+[S]}}$.

PEP-FOLD Simulation. PEP-FOLD is a computational tool used for predicting protein structure, specifically focusing on predicting the three-dimensional (3D) structure of peptides or small proteins [3, 4]. PEP-FOLD operates based on a de novo approach to structure prediction. The amino acid sequences were provided. PEP-FOLD then employs a physics-based energy function to calculate the energetically favorable conformations of the peptide chain in water. This involves considering various forces and interactions, such as bond angles, dihedral angles, and non-bonded interactions. PEP-FOLD generates a large number of potential conformations or structures for the given peptide sequence. This is often done using a Monte Carlo or molecular dynamics sampling approach. Each generated conformation is assigned a score based on its energy and the agreement with experimental or theoretical constraints. The algorithm selects the most energetically favorable conformations as potential predictions for the 3D structure of the peptide. Finally, PEP-FOLD provides the user with the predicted 3D structure(s) of the input peptide sequence. It is important to note that the accuracy of structure prediction tools can vary depending on the length and complexity of the peptide sequence.

Confocal Microscopy. Mammalian cells were seeded at a density of 1×10^6 cells/ mL in 300 µL of their respective medium in 35 mm glass-bottom dishes (Cellvis, Lot No.: D35-14-1.5-N) and allowed to grow overnight. The cells were subsequently treated with 100 µM of the prodrug-peptides for 3 hours at 37 °C. Next, cells were washed three times with sterile PBS. Mammalian cells were stained at a final concentration of 1X CellBrite Fix Membrane Stain 640 (Biotium) at 37 °C for 15 minutes. Cells were then washed 3 times by PBS and fixed by 4% paraformaldehyde in PBS (Thermo Scientific) at room temperature for 20 min. Cells were imaged by a confocal microscope (Leica SP8 with lighting deconvolution) at an excitation/ emission wavelength of 638 nm/ 667 nm.

Transmission Electron Microscopy. TEM images were taken using the JEOL 1200 EX II operated at 80 kV. The TEM grids were prepared by drop casting 2 µL of each sample followed by air drying overnight. Cellular morphology was conducted using the same microscope. Here, 70nm thin sections of plastic were embedded with the fixed samples to visualize cellular organization.

Mammalian cell culture. HEK293T cells were a kind gift from Professor Liangfang Zhang's nanomedicine lab (UCSD NanoEngineering). Cells were cultured in complete Dulbecco's Modified Eagle Medium (supplemented with 10% Fetal Bovine Serum, 1% Penicillin-Streptomycin), at 37 °C and 5% CO2. Passages were conducted with 0.25% Trypsin EDTA before experiments.

ROS Assay. The ROS H_2O_2 assay is a method used to measure the levels of hydrogen peroxide within cells. Hydrogen peroxide is a ROS that plays a crucial role in cellular signaling and oxidative stress. Upon addition of ROS-Glo™ Detection Reagent containing Ultra-Glo™ Recombinant Luciferase and d-Cysteine, the precursor is converted to luciferin by the d-Cysteine, and the produced luciferin reacts with Ultra-Glo™ Recombinant Luciferase to generate a luminescent signal that is proportional to H_2O_2 concentration. Cells were seeded into 96-well plates (Corning, white solid, CLS3570) at a density of 10^6 cells in 70 µL medium per well and incubated overnight. Next, 20 µL of combined H_2O_2 Substrate (Promega) and test compound to cells and mixed for a final well volume of 100 µL and the final H_2O_2 Substrate concentration of 25µM. The plate was

then incubated for 4 hours at 37 °C before 100µl of ROS-Glo™ Detection Solution was added to each well. Finally, the plate was incubated for 20 minutes at room temperature and the relative luminescence was recorded using a plate reader (Synergy H1, BioTek).

Limit of Detection. The limit of detection (LoD) was calculated using the limit of blank (LoB) as demonstrated by Armbruster et al [5]. The LoB uses a blank measurement to define the highest signal generated from the sample with no analyte. LoB was calculated using the mean ($mean_{blank}$) and standard deviation (SD_{blank}) of a blank sample:

$LoB = mean_{blank} + 1.645\ (SD_{blank})$

Based on this, the LoD is defined as the lowest analyte concentration that can be differentiated from the LoB. Here, the LoD represents an analyte concentration at which 95% of measured samples are readily differentiated from the LoB while the remaining 5% can contain no analyte:

$LoD = LoB + 1.645\ (SD_{low\ concentration\ sample})$.

Statistical Analysis. Statistical analysis was conducted using GraphPad Prism 10. Percent toxicity was quantified by averaging the absorbance for each repetition, and error bars represent the standard deviation across eight wells. For intra- and inter-assay variability, data from two consecutive experiments were analyzed. Percent total variation was calculated using ANOVA to compare viability before and after cleavage by recombinant prodrug. A P-value of < 0.05 was considered significant. The null hypotheses were: (1) There is no significant difference in viability among cell types. (2) There is no significant difference in viability among treatments. (3) There is no interaction effect between cell type and treatment on viability. Multiple Unpaired Student's t tests were also performed to compare the significance between variables.

II. Supplementary Data

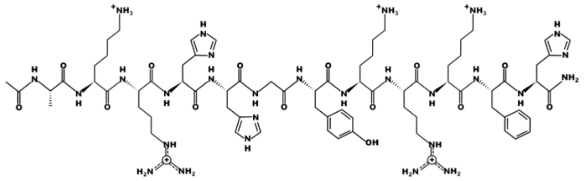

P-113: AKRHHGYKRKFH (1604.8891 g/mol)

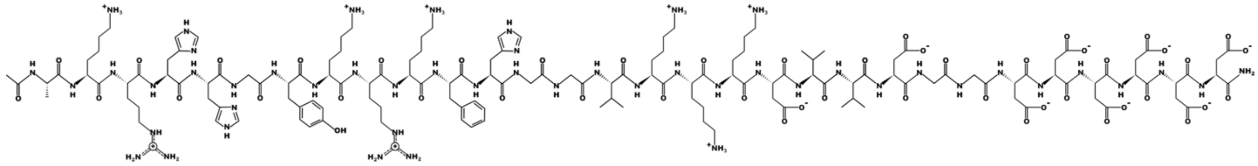

S1: AKRHHGYKRKFHGGVKKKDVVDGGDDDDDD (3434.6778 g/mol)

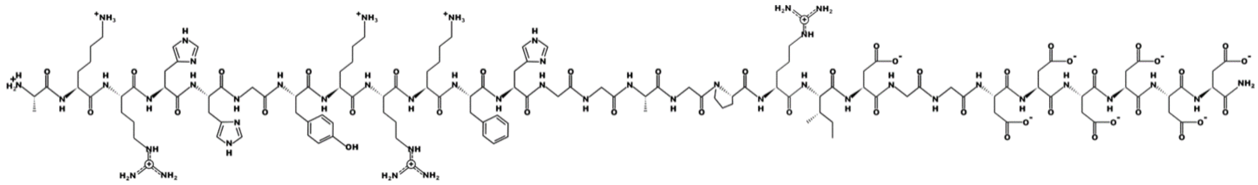

G1: AKRHHGYKRKFHGGAGPRIDGGDDDDDD (3090.4474 g/mol)

Figure S1. Structure of P-113 and SAP9 and RgpB- cleavable prodrugs, S1 and G1, respectively (top to bottom).

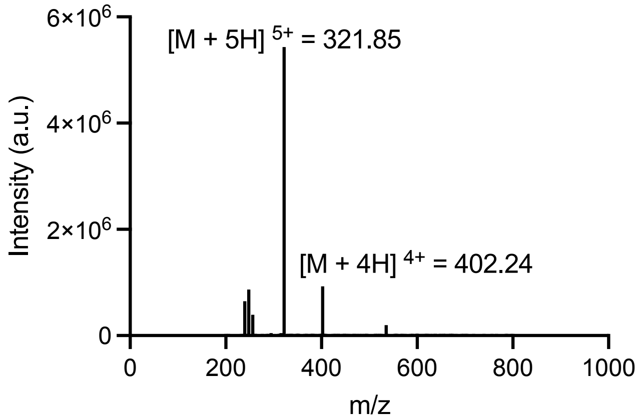

Figure S2. P-113 characterization. ESI-MS with theoretical mass: 1604.8891 Da, $[M + 5H]^{5+}$ = 321.98 m/z; $[M + 4H]^{4+}$ = 402.22 m/z.

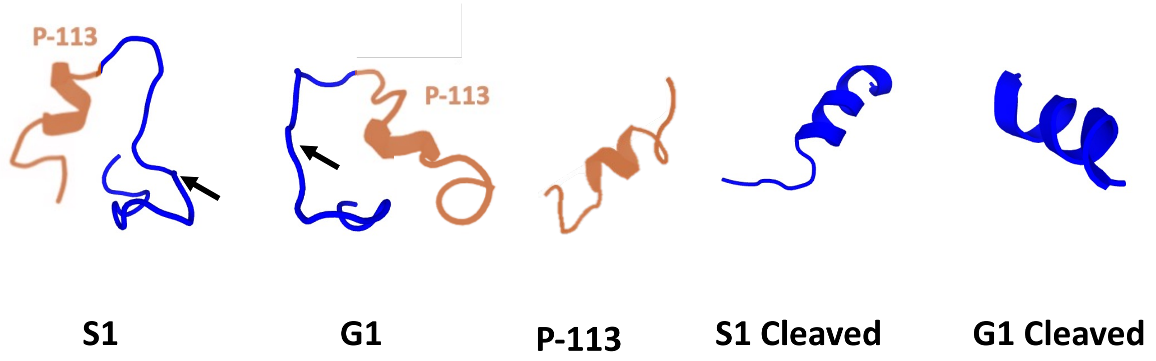

Figure S3. Simulated secondary structure of prodrugs. S1 and G1 both show the intact coil structure of P-113 and the cleavage site as shown by the arrow. Red highlights the antimicrobial fragment of the prodrugs. P-113 was also simulated to confirm literature-based analyses of the structure [6]. Finally, the secondary structure of the cleaved prodrugs was simulated and are shown to mimic the shape of P-113.

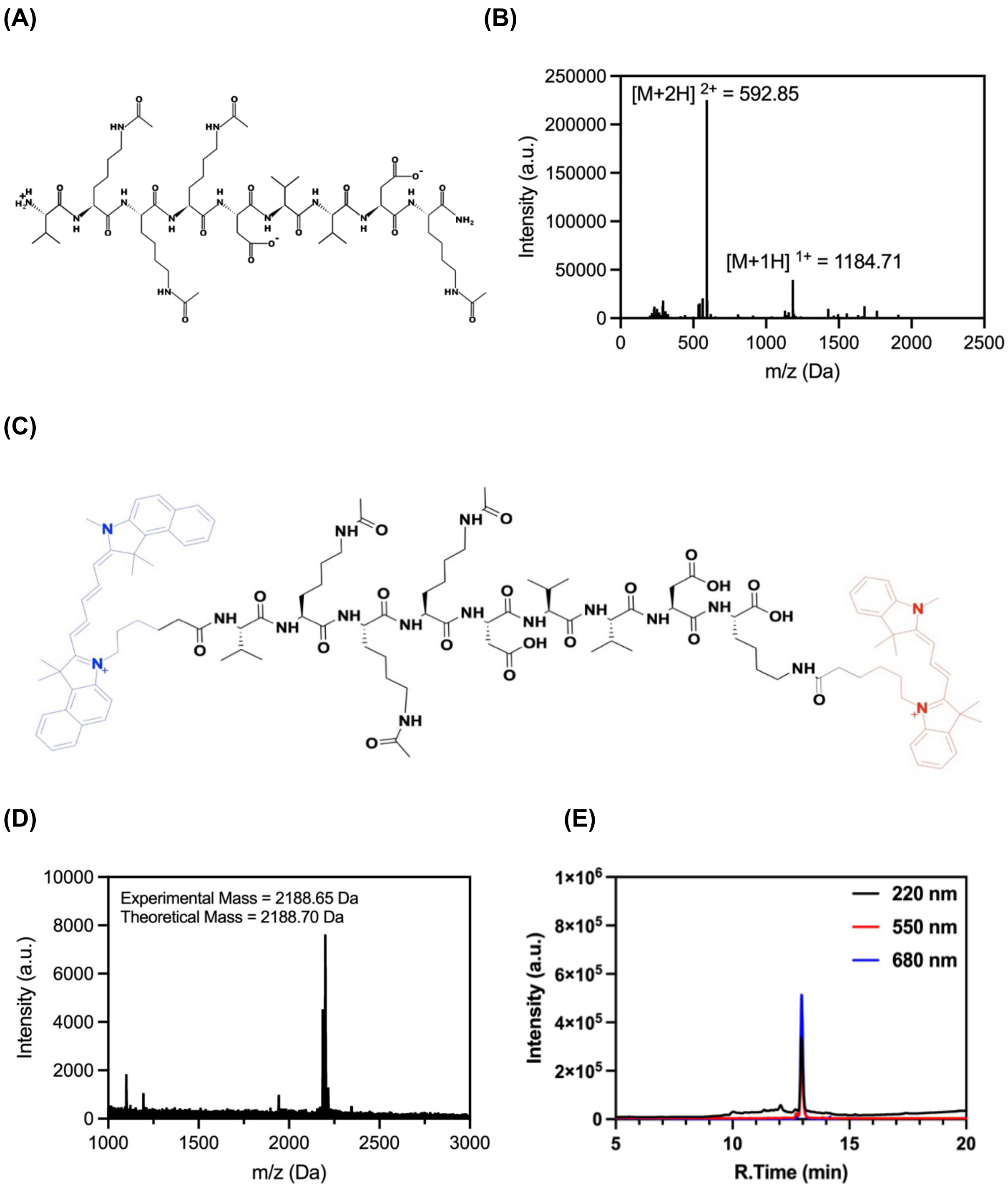

Figure S4. SAP9 cleavable substrate FRET probe characterization. (A) SAP9 cleavable substrate peptide structure: Val- aLys- aLys- aLys- Asp- Val -Val -Lys with theoretical mass 1182.6953 Da. **(B)** ESI-MS presenting two clear peaks at $[M+2H]^{2+}$ = 592.34765 and $[M+1H]^{1+}$ = 1183.6953. **(C)** Structure of SAP9 cleavable probe with conjugated NHS-dyes on either terminal with theoretical mass 2188.8039 Da. **(D)** MALDI-TOF spectra **(E)** HPLC spectra monitoring at peptide bond wavelength, 220 nm, and cyanine dye wavelengths, 550 and 680 nm confirming successful conjugation.

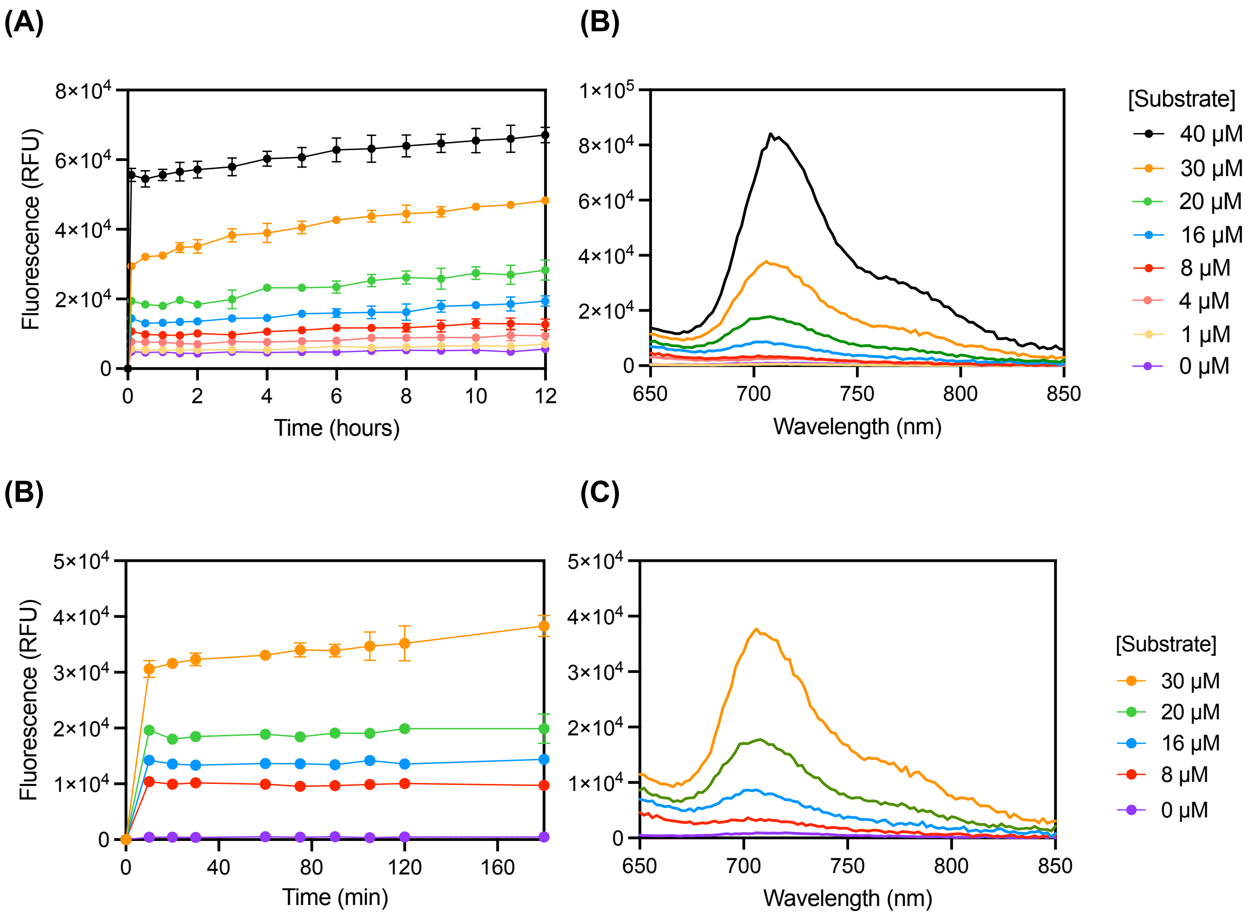

Figure S5. SAP9 cleavable substrate FRET probe fluorescence. (A) Kinetic data collected over 12-hour period. **(B)** Total corresponding fluorescence emission spectra (Ex.620 nm) after 3 hours incubation at 37°C at increasing concentration of substrate. **(C)** Extracted data for 3-hour incubation demonstrating maximum fluorescence reached. **(D)** Zoomed in (0 – 30 μM) corresponding fluorescence emission spectra (Ex.620 nm) after 3 hours incubation at 37°C at increasing concentration of substrate.

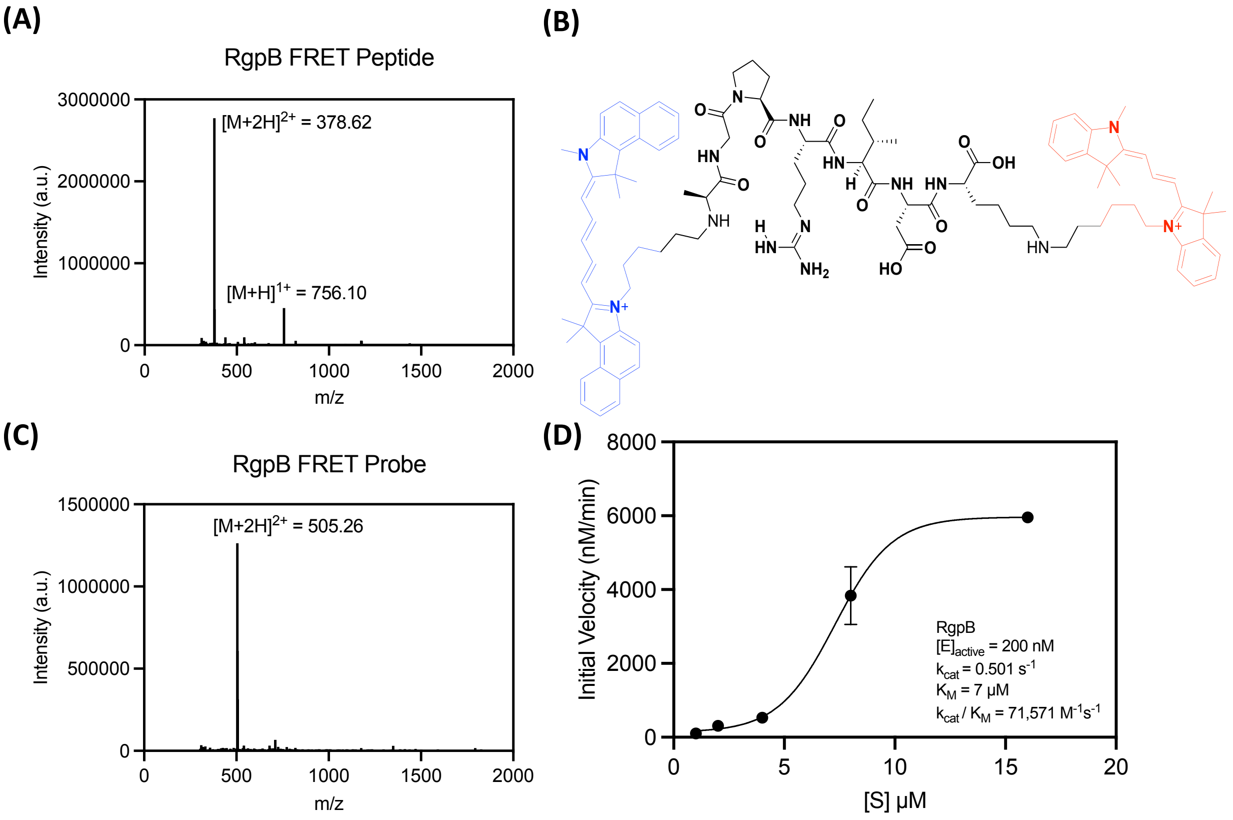

Figure S6. RgpB cleavable substrate FRET probe characterization. (A) RgpB cleavable substrate peptide structure: Ala – Gly – Pro – Arg – Ile – Asp – Lys. ESI-MS presenting two clear peaks at $[M+2H]^{2+}$ = 378.62 and $[M+1H]^{1+}$ = 756.10. **(B)** Structure of RgpB cleavable probe with conjugated NHS-dyes on either terminal with theoretical mass 1513.2932 Da. **(C)** ESI-MS spectra with clear peak at $[M+2H]^{2+}$ = 505.26 confirming successful conjugation. **(D)** Michaelis-Menten master curve describing the rate of reaction with an increase in substrate concentration to determine kcat/ KM, the second-order rate constant reaction rate of the enzyme-substrate complex to product. Here, a higher ratio suggests a higher rate of conversion. Inset parameters present: [E]active, the active enzyme concentration used, 200 nM. Catalytic constant, kcat, the number of probe molecules converted by enzyme per second. The Michaelis-Menten constant, KM, an inverse measurement of affinity.

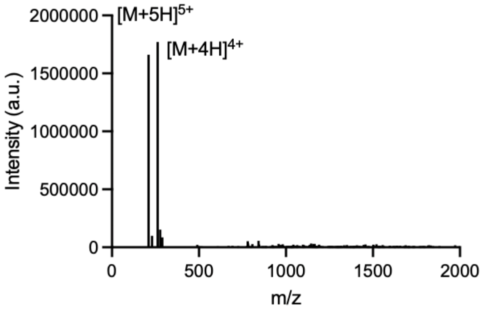

Figure S7. ESI-MS characterization of purified fragment corresponding to G1 toxicity blocker. Two distinct peaks at $[M+5H]^{5+}$ =210.23 and $[M+4H]^{4+}$= 262.26 for the theoretical mass of the G1 toxicity blocker fragment 1047.3407.

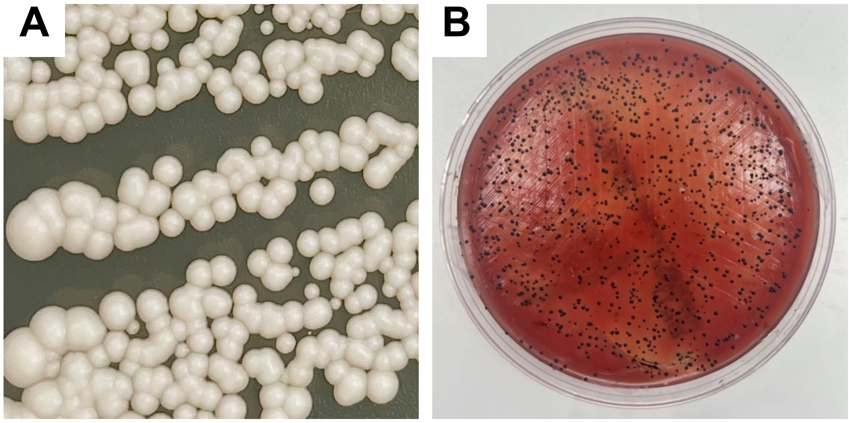

Figure S8. Microorganism growth for disk diffusion test. (A) String of pearls" characteristic of *C. albicans* formation. **(B)** *P. gingivalis* colonies with characteristic black pigmentation plated and grown anaerobically on blood agar.

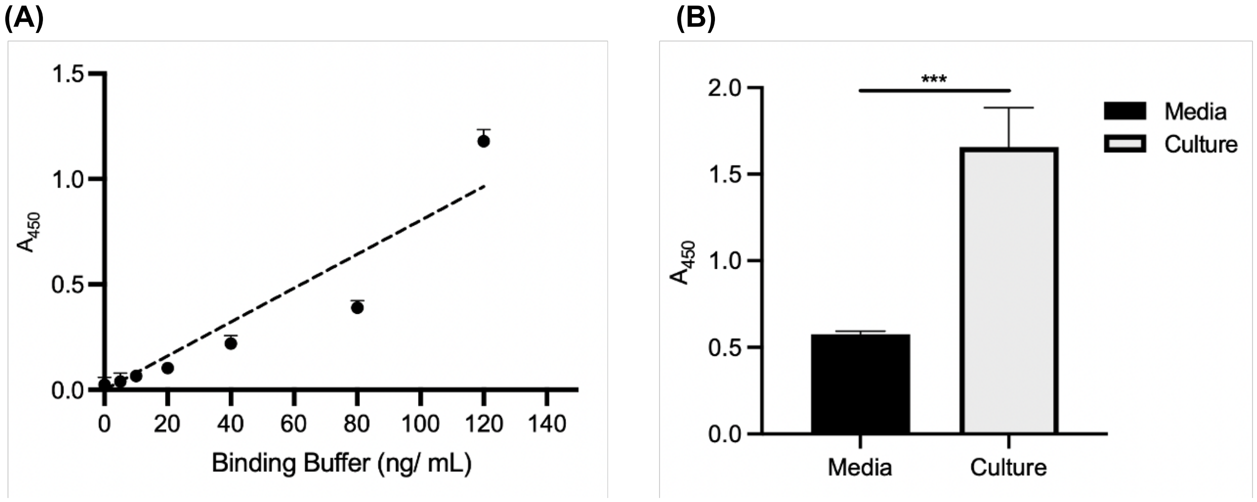

Figure S9. ELISA detection of SAP in *C. albicans* culture. (A) Standard curve for SAP detected via commercially available ELISA where m = $6.8 \times 10^{-3} \pm 0.1 \times 10^{-4}$. **(B)** C.a. culture (OD = 0.5) contained significantly more SAP than yeast peptone dextrose media alone. [SAP] from cells = 243 ± 33 ng/mL. Error bars represent the standard error for the mean for n = 6. Asterisks denote values from a one-tailed t-test (***$p < 0.0001$).

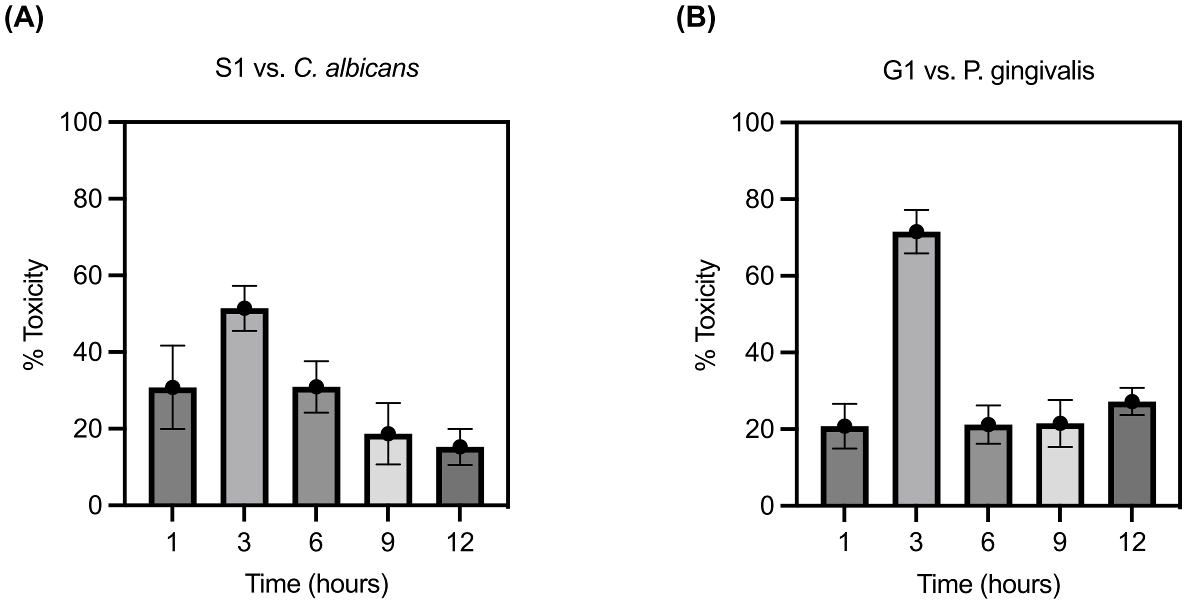

Figure S10. Time dependent toxicity assessment for S1 and G1. (A) XTT viability assay for *C. albicans* cells incubated with 10 μM of S1 in MES buffer over time. Here, an increased toxicity is observed at 3 hours. **(B)** XTT viability assay for *P. gingivalis* cells incubated with 10 μM of G1 in Tris buffer over time. Again, an increased toxicity is observed at 3 hours. Cells were washed with PBS to prevent continued antimicrobial activity past the desired time.

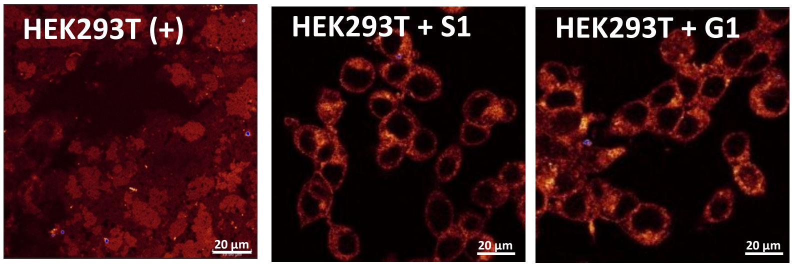

Figure S11. Confocal microscopy of mammalian cells. HEK293T demonstrating a deconstructed cell membrane when treated with bleach (positive control) intact cell membrane after treatment with prodrugs S1 and G1 for 3 hours at 37 °C. Cells were stained at a final concentration of 1X at 37 °C for 15 minutes with CellBrite Fix Membrane Stain 640, fixed with 4% paraformaldehyde, and imaged with a Leica SP8 with lighting deconvolution at an excitation/ emission wavelength of 638 nm/ 667 nm.

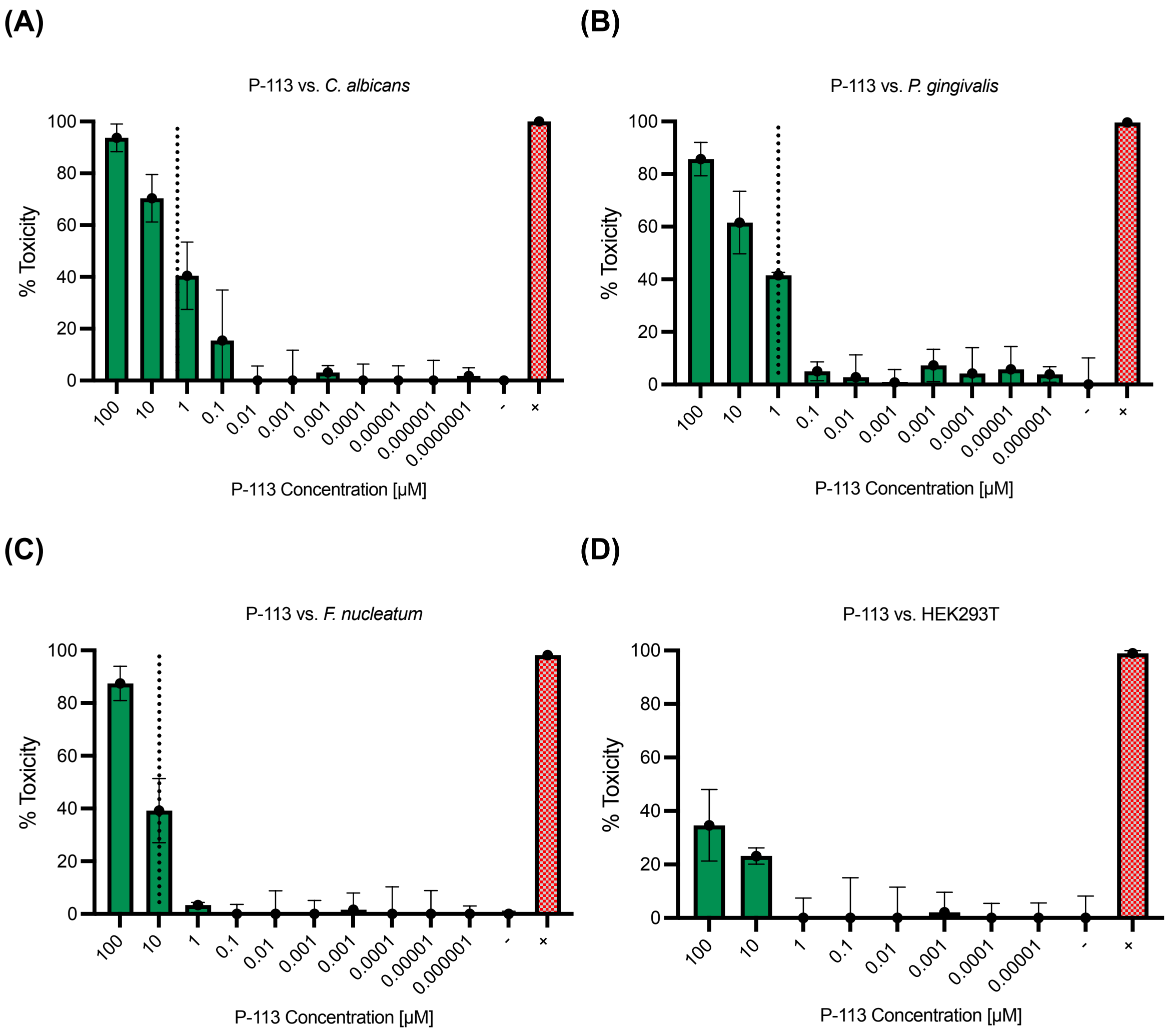

Figure S12. Toxicity assessment for varying concentrations of P-113, positive control, treatment along four cell lines. (A) *C. albicans* with negative control (-) MES buffer and positive control (+) Fluconazole. **(B)** *P. gingivalis* with negative control (-) Tris buffer and positive control (+) Penicillin-streptomycin. **(C)** *F. nucleatum* with negative control (-) Tris buffer and positive control (+) Penicillin-streptomycin. **(D)** HEK293T with negative control (-) PBS and positive control (+) 10% bleach. Dotted line represents MIC as the lowest concentration that results in a significant decrease in toxicity.

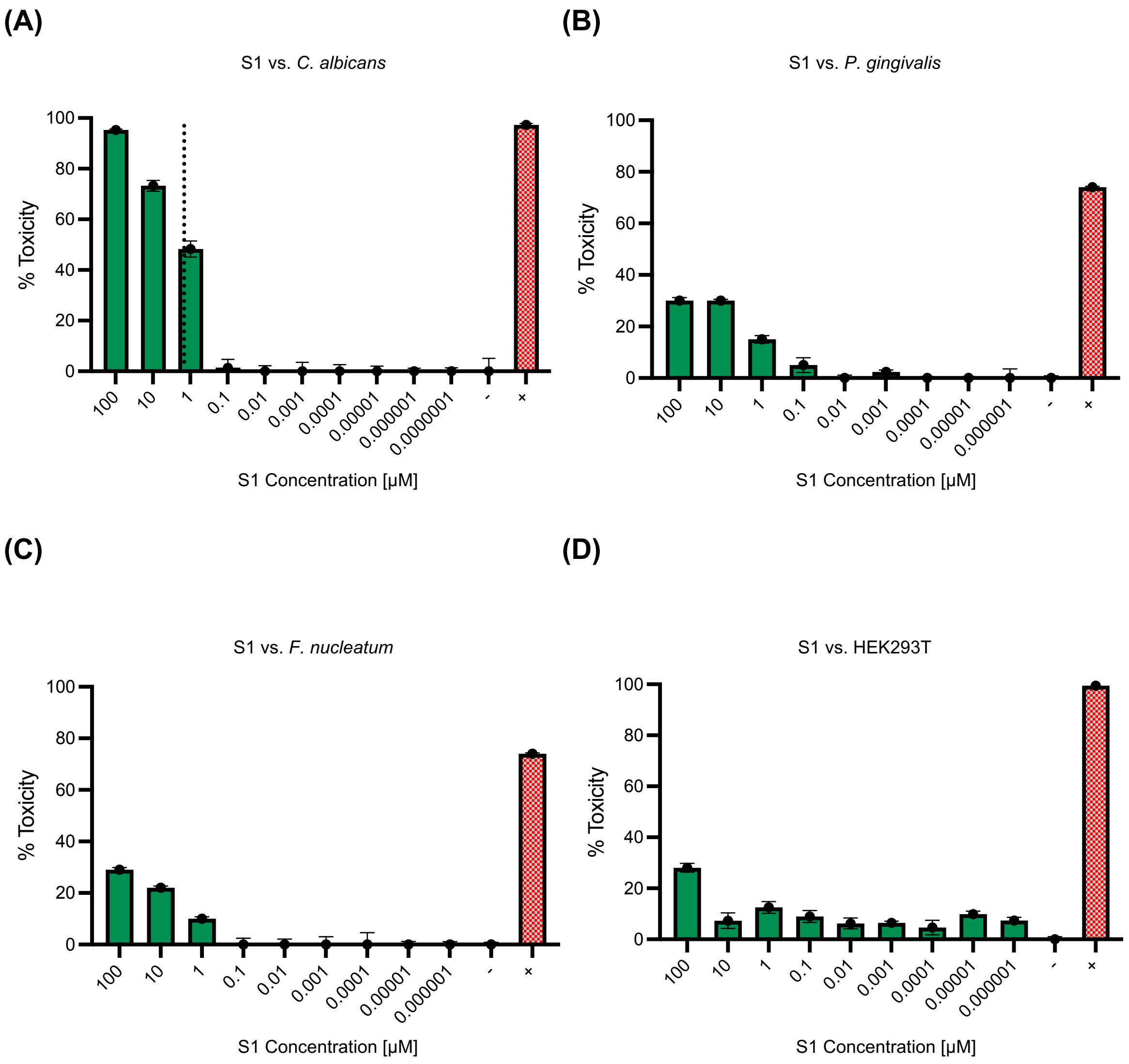

Figure S13. Toxicity assessment for varying concentrations of S1, *C. albicans* prodrug, treatment, along four cell lines. (A) *C. albicans* with negative control (-) MES buffer and positive control (+) Fluconazole. **(B)** *P. gingivalis* with negative control (-) Tris buffer and positive control (+) Penicillin-streptomycin. **(C)** *F. nucleatum* with negative control (-) Tris buffer and positive control (+) Penicillin-streptomycin. **(D)** HEK293T with negative control (-) PBS and positive control (+) 10% bleach. Dotted line represents MIC as the lowest concentration that results in a significant decrease in toxicity.

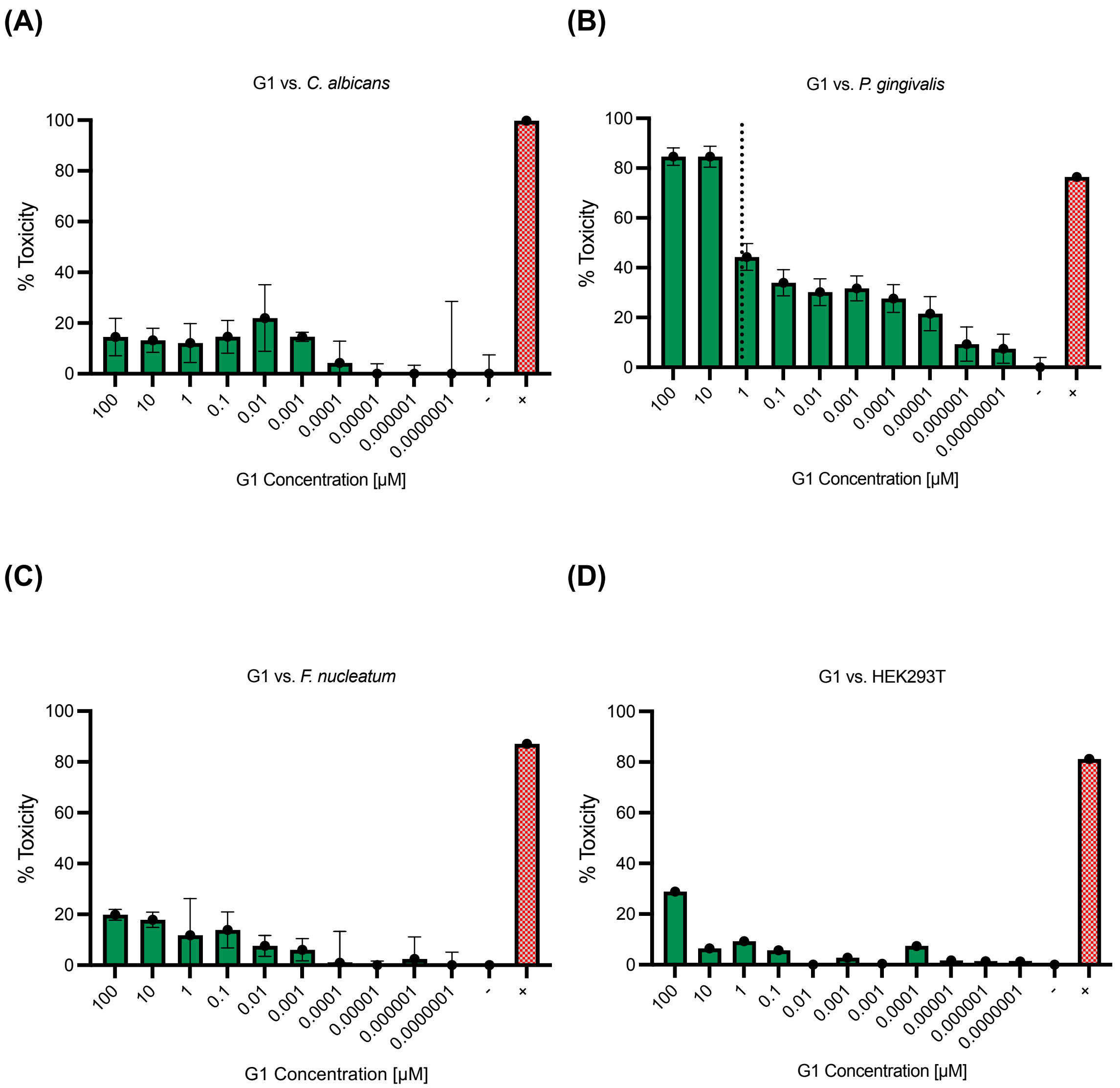

Figure S14. Toxicity assessment for varying concentrations of G1, *P. gingivalis* prodrug, treatment, along four cell lines. (A) *C. albicans* with negative control (-) MES buffer and positive control (+) Fluconazole*.* **(B)** *P. gingivalis* with negative control (-) Tris buffer and positive control (+) Penicillin-streptomycin*.* **(C)** *F. nucleatum* with negative control (-) Tris buffer and positive control (+) Penicillin-streptomycin. **(D)** HEK293T with negative control (-) PBS and positive control (+) 10% bleach. Dotted line represents MIC as the lowest concentration that results in a significant decrease in toxicity.

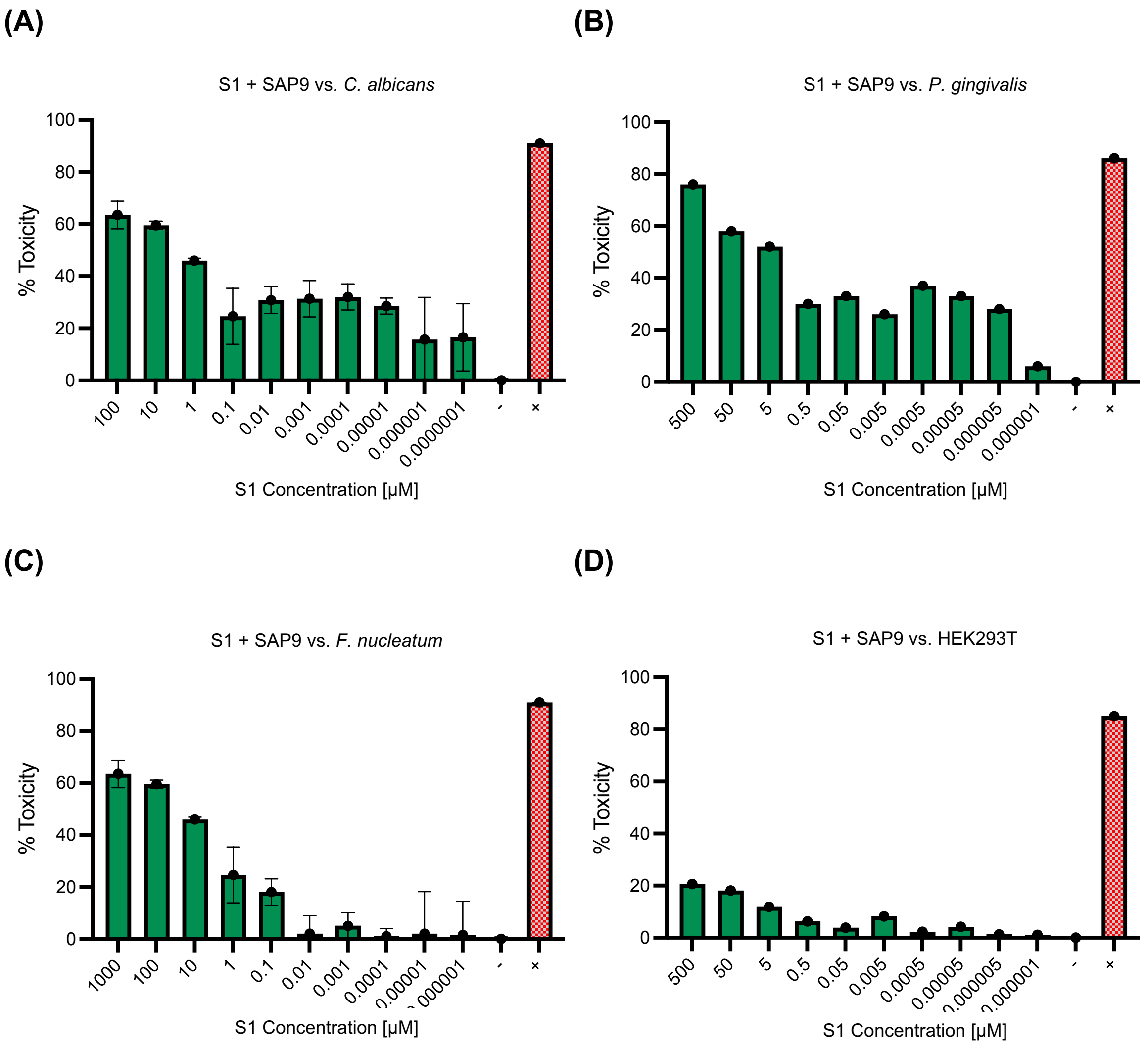

Figure S15. Toxicity assessment for varying concentrations of S1 + SAP9, *C. albicans* prodrug pre-cleaved by 200 nM recombinant protease in vitro, treatment, along four cell lines. (A) *C. albicans* with negative control (-) MES buffer and positive control (+) Fluconazole. **(B)** *P. gingivalis* with negative control (-) Tris buffer and positive control (+) Penicillin-streptomycin. **(C)** *F. nucleatum* with negative control (-) Tris buffer and positive control (+) Penicillin-streptomycin. **(D)** HEK293T with negative control (-) PBS and positive control (+) 10% bleach.

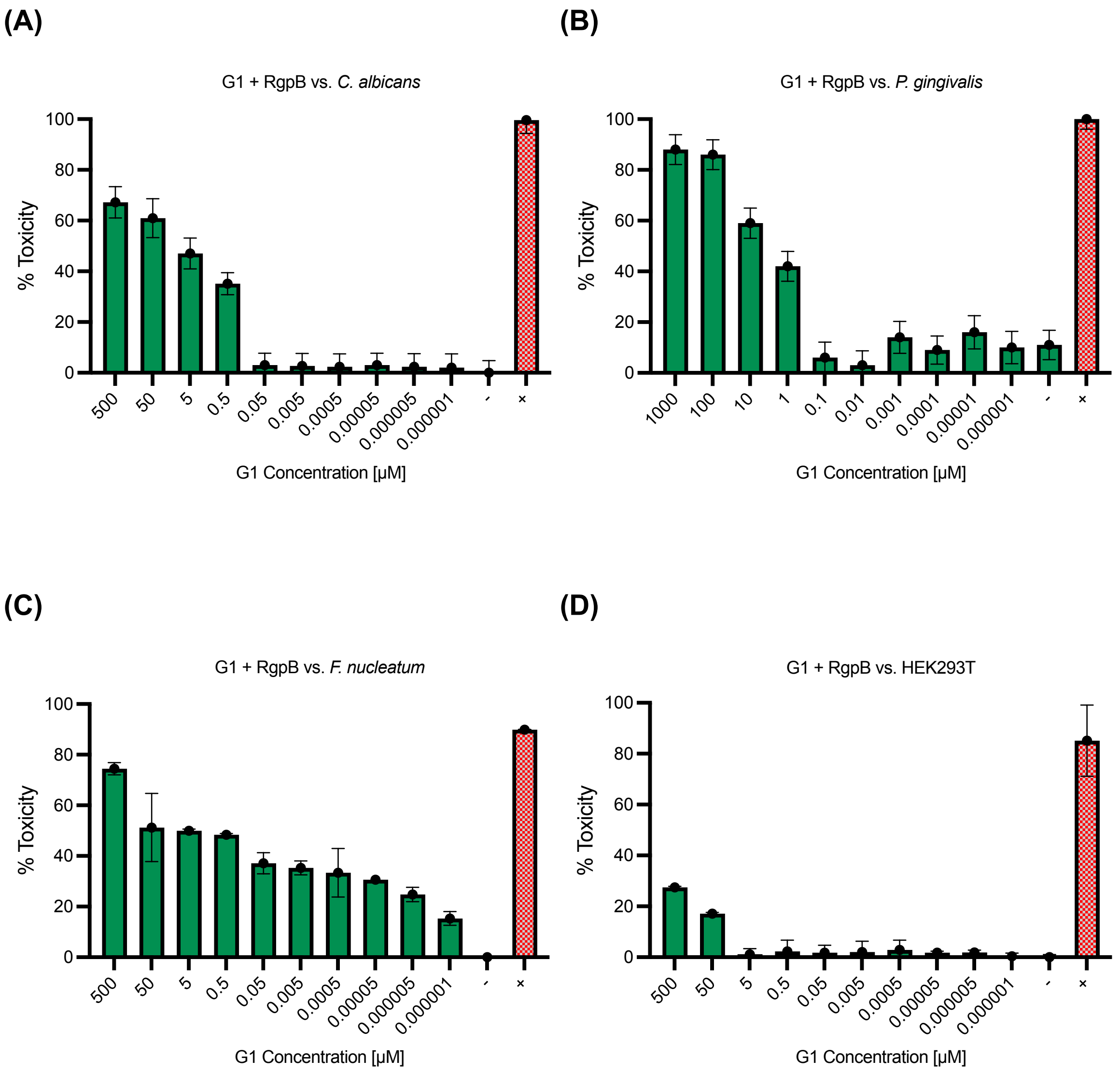

Figure S16. Toxicity assessment for varying concentrations of G1 + RgpB, *P. gingivalis* prodrug pre-cleaved by 200 nM recombinant protease in vitro, treatment, along four cell lines. (A) *C. albicans* with negative control (-) MES buffer and positive control (+) Fluconazole. **(B)** *P. gingivalis* with negative control (-) Tris buffer and positive control (+) Penicillin-streptomycin. **(C)** *F. nucleatum* with negative control (-) Tris buffer and positive control (+) Penicillin-streptomycin. **(D)** HEK293T with negative control (-) PBS and positive control (+) 10% bleach.

C. albicans vs. HEK293T

Treatment	P value	Significant
P-113	0.000065	Yes
PBS	>0.999999	No
S1	<0.000001	Yes
G1	0.028335	No

P. gingivalis vs. HEK293T

Treatment	P value	Significant
P-113	0.000747	Yes
PBS	>0.999999	No
G1	<0.000001	Yes

C. albicans vs. *P. gingivalis*

Treatment	P value	Significant
P-113	0.284304	No
PBS	>0.999999	No
S1	<0.000001	Yes
G1	<0.000001	Yes

Figure S17. Statistical analysis to highlight the antimicrobial specificity of protease-activated prodrugs. A two-way ANOVA was performed with the cell type and treatment as independent variables and viability as the dependent variable. The null hypotheses are: (1) There is no significant difference in viability among cell types and (2) There is no significant difference in viability among treatments. (3) There is no interaction effect between cell type and treatment on viability. The calculated P-value for all three was <0.0001 suggesting significant data and as such ($P < 0.05$), a statistically significant difference in viability between cell types, a difference in viability and an effect between cell type and treatment. Eight replicates were used which resulted in an average statistical power of 0.9 when $\alpha = 0.05$. Thus, there is a strong chance of correctly rejecting the null hypothesis if there is a true effect under these experimental parameters.

	**Peptidase**
P. gingivalis	granzyme B ({Homo sapiens}-type)
	meprin alpha subunit
	meprin beta subunit
	legumain, animal-type
C. albicans	granzyme B ({Homo sapiens}-type)
	meprin alpha subunit
	meprin beta subunit
	legumain, animal-type

Figure S18. MEROPS Analysis. Proteases secreted by the microorganisms that cleave an aspartic acid substrate bond.

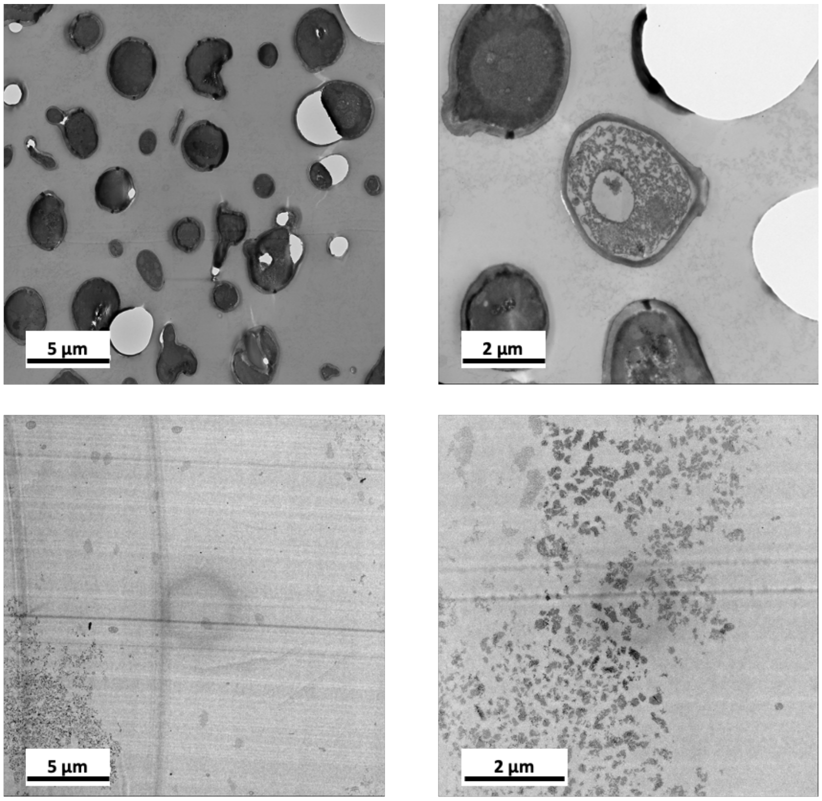

Figure S19. ***C. albicans*** **TEM images of cellular morphology at increased magnification.** Top: Untreated cells showing intact cell membranes at 5 µm and 2 µm scale bars (left to right). Bottom: Treated cells with S1 for 3 hours at 37 °C showing deconstructed cell structure and no evident cell membrane or organism.

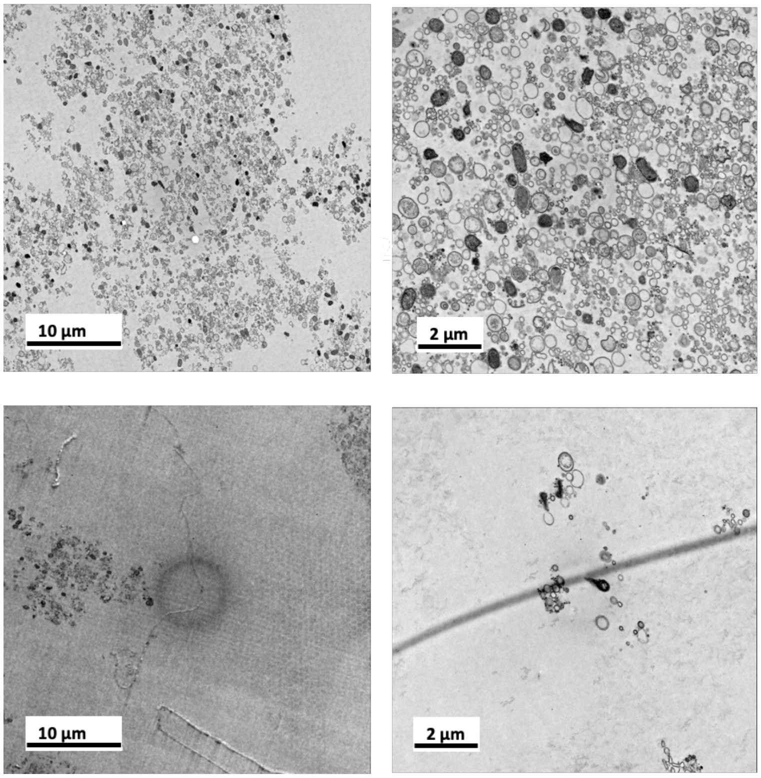

Figure S20. ***P. gingivalis*** **TEM images of cellular morphology at increased magnification.** Top: Untreated cells showing intact cell membranes at 10 µm and 2 µm scale bars (left to right). Bottom: Treated cells with G1 for 3 hours at 37 °C showing notable decrease in cell density and destruction of cell structure.

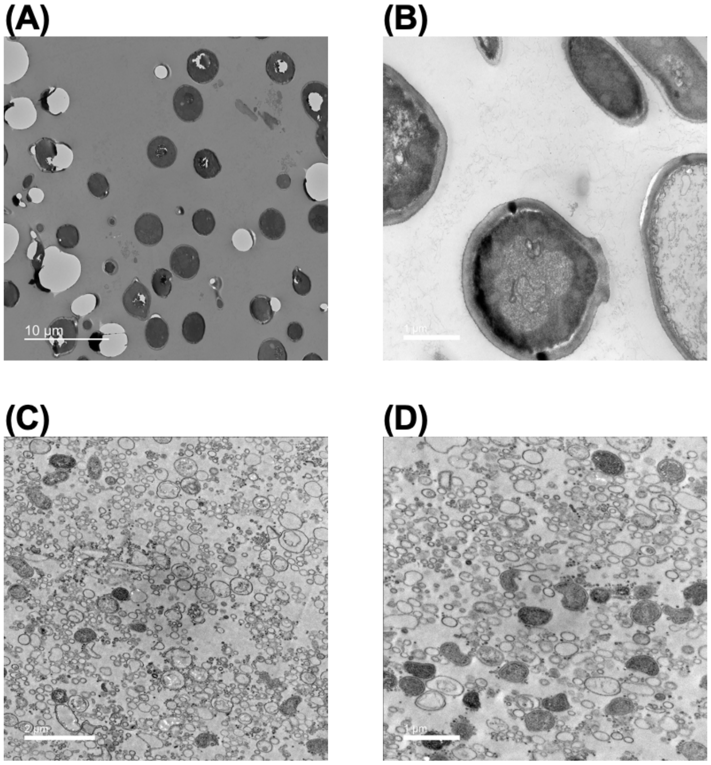

Figure S21. Intact prodrug effects on membrane integrity. (A, B) *C. albicans* TEM images of cellular morphology after treatment with G1 for 3 hours at 37 °C; there are intact cell structures and cell membranes. Panel A scale bar is 10 µm and B is 1 µm. (C, D) *P. gingivalis* TEM images of cellular morphology after treatment with S1 for 3 hours at 37 °C showing intact cell structures and membranes. Panel C scale bar is 2 µm and D is 1 µm.

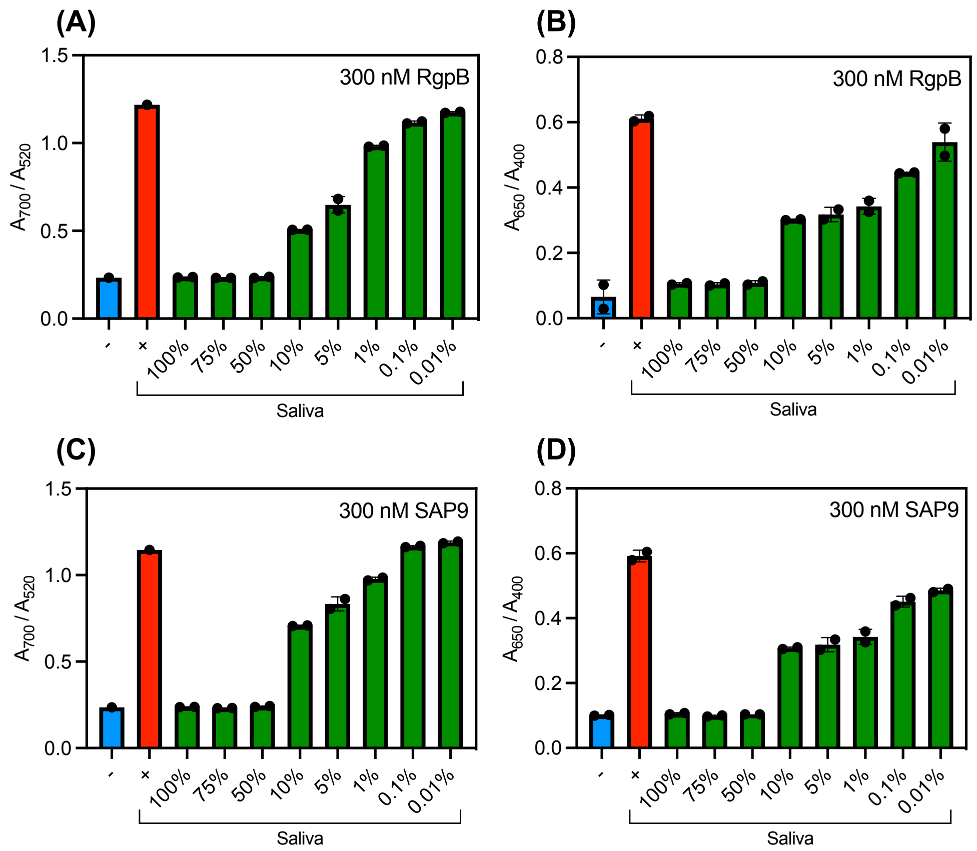

Figure S22. Protease-induced plasmonic nanoparticle assembly in saliva. (A) Optical absorption of AuNPs and **(B)** AgNPs when incubated with increased concentrations of saliva spiked with 300 nM RgpB in activity buffer at constant G1 concentration (1:1000, E:P). **(C)** Optical absorption of AuNPs and **(D)** AgNPs when incubated with increased concentrations of saliva spiked with 300 nM SAP9 in activity buffer at constant S1 concentration (1:1000, E:P). Negative control (-) uses monodispersed nanoparticles in activity buffer and positive control (+) is 0% saliva (i.e., 100% activity buffer).

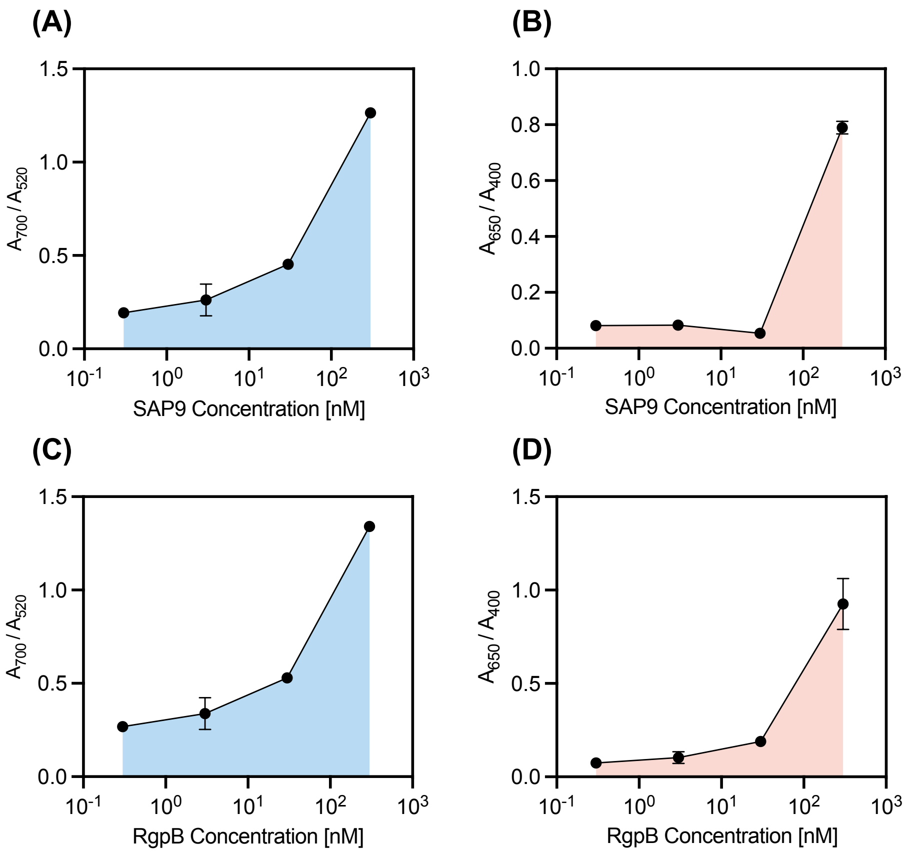

Figure S23. Limit of detection of plasmonic nanoparticle assembly in saliva. (A) Limit of detection (LOD) for AuNP and **(B)** AgNP detection system of S1 using an increasing concentration of SAP9. **(C)** Limit of detection (LOD) for AuNP and **(B)** AgNP detection system of G1 using an increasing concentration of RgpB. LOD study shows an increase in the limit for both proteases in complex media.

(A) (B)

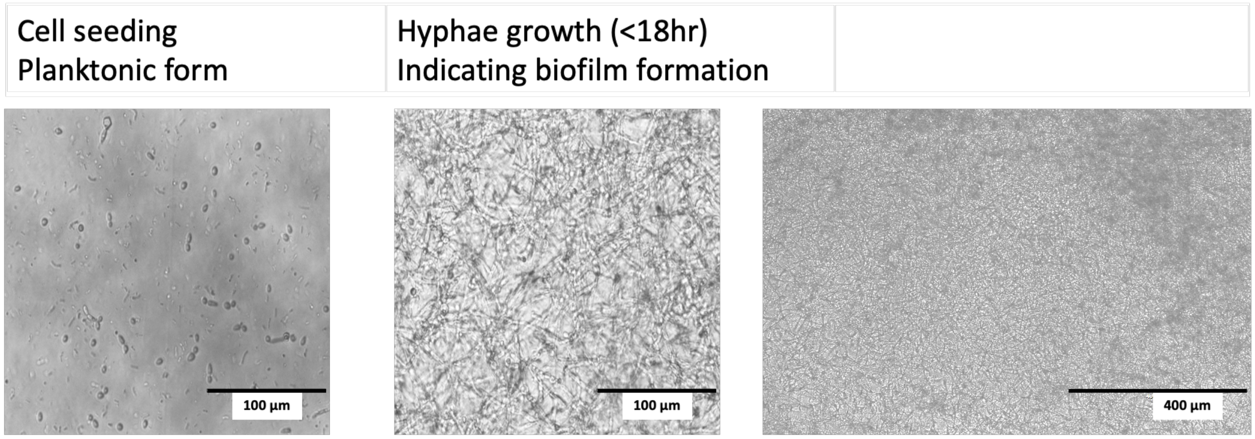

Figure S24. *C. albicans* growth transmission microscopy. (A) *C. albicans* cell seeding planktonic form (100 µm) and **(B)** hyphae growth (<18 hr.) indicating biofilm formation after 18 hours at 30°C (100 µm) and expanded to 400 µm.

III. Supplementary References

1. Retout M, Amer L, Yim W, Creyer MN, Lam B, Trujillo DF, et al. A protease-responsive polymer/peptide conjugate and reversible assembly of silver clusters for the detection of Porphyromonas gingivalis enzymatic activity. ACS Nano. 2023; 17:17308-17319.
2. Yim W, Retout M, Chen AA, Ling C, Amer L, Jin Z, et al. Goldilocks energy minimum: Peptide-based reversible aggregation and biosensing. ACS Appl Mater Interfaces. 2023; 15: 42293-303.
3. Alland C MF, Boens D, Carpentier M, Chiusa S, Lonquety M, Renault N, et al. PEP-FOLD RPBS: A web resource for structural bioinformatics. Nucleic Acids Res; 2005.
4. Néron B MH, Maufrais C, Joly N, Maupetit J, Letort S, Carrere S, et al. Mobyle: A new full web bioinformatics framework. Bioinformatics: Epub 2009.
5. Armbruster DA, Pry T. Limit of blank, limit of detection and limit of quantitation. Clin Biochem Rev. 2008; 29 Suppl 1: S49-52.
6. Tsai C-Y, Salawu EO, Li H, Lin G-Y, Kuo T-Y, Voon L, et al. Helical structure motifs made searchable for functional peptide design. Nat Commun. 2022; 13: 102.

IV. Author Contributions

L.A. proposed the system, designed, and conducted experiments, led data curation and analysis, and wrote the manuscript. M.R. helped design the system, conduct experiments, and analyze data. J.V.J. led project administration, and conceived, and supervised the work. All authors edited the manuscript.
